# Angiogenic Microvascular Wall Shear Stress Patterns Revealed Through Three-dimensional Red Blood Cell Resolved Modeling

**DOI:** 10.1093/function/zqad046

**Published:** 2023-08-29

**Authors:** Mir Md Nasim Hossain, Nien-Wen Hu, Maram Abdelhamid, Simerpreet Singh, Walter L Murfee, Peter Balogh

**Affiliations:** Mechanical and Industrial Engineering, New Jersey Institute of Technology, Newark, NJ 07114, USA; J. Crayton Pruitt Family Department of Biomedical Engineering, University of Florida, Gainesville, FL 32611, USA; Mechanical and Industrial Engineering, New Jersey Institute of Technology, Newark, NJ 07114, USA; Mechanical and Industrial Engineering, New Jersey Institute of Technology, Newark, NJ 07114, USA; J. Crayton Pruitt Family Department of Biomedical Engineering, University of Florida, Gainesville, FL 32611, USA; Mechanical and Industrial Engineering, New Jersey Institute of Technology, Newark, NJ 07114, USA

**Keywords:** angiogenesis, microcirculation, wall shear stress, red blood cells

## Abstract

The wall shear stress (WSS) exerted by blood flowing through microvascular capillaries is an established driver of new blood vessel growth, or angiogenesis. Such adaptations are central to many physiological processes in both health and disease, yet three-dimensional (3D) WSS characteristics in real angiogenic microvascular networks are largely unknown. This marks a major knowledge gap because angiogenesis, naturally, is a 3D process. To advance current understanding, we model 3D red blood cells (RBCs) flowing through rat angiogenic microvascular networks using state-of-the-art simulation. The high-resolution fluid dynamics reveal 3D WSS patterns occurring at sub-endothelial cell (EC) scales that derive from distinct angiogenic morphologies, including microvascular loops and vessel tortuosity. We identify the existence of WSS hot and cold spots caused by angiogenic surface shapes and RBCs, and notably enhancement of low WSS regions by RBCs. Spatiotemporal characteristics further reveal how fluctuations follow timescales of RBC “footprints.” Altogether, this work provides a new conceptual framework for understanding how shear stress might regulate EC dynamics in vivo.

## Graphical Abstract

**Graphical Abstract:**
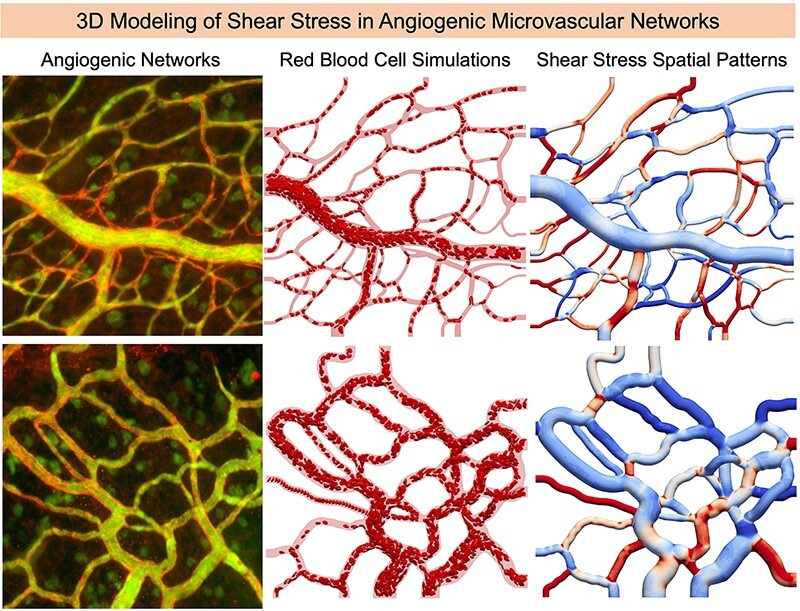

## Introduction

Angiogenesis, the process of growing new blood vessels from existing vessels, generally occurs in response to local stimuli. Predominant among these is the wall shear stress (WSS) exerted by blood flowing through microvascular capillary vessels.^1,2^ Endothelial cells (ECs) line blood vessel walls and can sense and respond to external forces leading to new vessel sprouts, growth through tissue, and formation of highly complex vessel networks.^3–5^ Three-dimensional (3D) WSS characteristics experienced due to red blood cells (RBCs) flowing through real angiogenic microvascular networks with complex surface topologies, however, are generally unknown. Making such measurements in experiments is difficult, and in silico modeling that resolves the necessary 3D geometric details and explicit RBC deformation is extremely challenging. Studies have shown intricate WSS patterns can arise due to complexities such as EC curvature^6^ and 3D shapes,^7^ and more recently due to complex 3D vessel morphologies and influence of RBCs.^8^ Such findings underscore the importance of elucidating 3D WSS complexities that occur during angiogenesis and the associated microvascular network remodeling in vivo, knowledge of which can help researchers build a new mechanistic understanding of the processes in 3D leading to profound impacts affecting public health.

Angiogenic processes are ubiquitous at all stages of animal life, and in both health and disease.^9^ The development and growth of organs in embryos is facilitated by angiogenesis,^10^ and continues up until adulthood.^9^ Processes associated with wound healing,^11–13^ the menstrual cycle,^14,15^ and pregnancy^16,17^ involve angiogenesis, as do exercise^18^ and muscle adaptation.^19,20^ The development and progression of many diseases also rely on angiogenesis.^21^ In cancer, the process enables invasion of tumors into tissue and can even supply a route for metastatic cells to exit and circulate to other regions of the body.^9^ In diabetes, decreased angiogenesis can lead to impaired wound healing^22,23^ and abnormal blood vessel growth in the retina can lead to blindness.^24–26^ In ischemic heart disease, the body uses angiogenesis to improve blood flow near vascular occlusions and other regions in need.^27–29^ Other conditions such as psoriasis,^30,31^ arthritis,^32,33^ osteoporosis,^34,35^ and macular degeneration^36,37^ all involve angiogenesis as well.

Current knowledge of hemodynamic characteristics that exist during angiogenesis in vivo has in large part been provided by in silico fluid dynamics modeling. For example, WSS variations around isolated microvessel loop structures in avian chick embryos were shown using a 2D single-phase blood model, and connections between specific WSS patterns and sprout locations were identified.^38^ Other work in retinal angiogenic networks using a 3D single-phase flow model demonstrated general WSS values, which can lead to vessel regression.^39^ Modeling approaches have also integrated hemodynamics with tissue-side factors to further elucidate mechanistic roles driving network growth and adaptation, by considering vessels as either 2D^40,41^ or 1D lumped segments.^42–44^ Beyond these studies a wide variety of multiphysics modeling paradigms exist as well.^45–55^ While much has been learned regarding the WSS behavior associated with angiogenic events, 3D aspects and influences of RBCs are still unclear.

Direct evidence that ECs respond to applied forces has mainly come from experiments focusing on isolated ECs. Specific WSS values eliciting EC responses in vitro, commonly measured in parallel plate flow-type devices, have been reported to range anywhere from 0.1 to 100 dyne/cm.^21,5^,^56–60^ While such findings importantly establish physiological context for WSS magnitudes and angiogenic behavior, the significant range of WSS values spanning three orders of magnitude leads to questions concerning what is actually experienced by ECs during angiogenesis in vivo. Such questions are further complicated by findings from other in vitro works that have demonstrated strong EC responses to WSS spatial patterns or gradients,^61–64^ as opposed to just single WSS values. A few recent in vivo works have demonstrated WSS values up to 2 dyne/cm^2^ corresponding to EC rearrangement in developing retinal networks,^65^ or during intussusceptive angiogenesis in ischemic muscle.^66^ Yet, what do 3D angiogenic WSS patterns look like in vivo? This seemingly simple question represents a major gap in understanding because new vessel growth and adaptation occurs in vivo through 3D changes to complex surface morphologies, not as a 1D or 2D process or in response to a single WSS value. Moreover, since microcirculatory vessel diameters are similar to or smaller than individual RBC size,^67,68^ RBC deformation and interactions coupled with vessel surface complexity strongly influence the fluid dynamics and near-wall behavior, as has been shown by recent high-resolution modeling.^8,69^ This further emphasizes the importance of revealing 3D angiogenic WSS patterns because angiogenic microvascular networks have distinct geometric features such as looping vessel structures,^38^ frequent branching patterns,^70^ and increased vessel tortuosity.^71^

Toward this end, we reveal and quantify in this work 3D WSS characteristics that occur in real angiogenic microvascular networks by integrating high-fidelity RBC-resolved simulations with imaging of the rat mesentery undergoing angiogenesis. Specifically, we study the effects of angiogenic remodeling on local WSS distributions that in-turn influence EC responses. Behavior is identified by first focusing on variations from vessel to vessel to establish context for describing highly localized 3D variations in both space and time in subsequent sections. Detailed maps of WSS spatial patterns are provided, which illustrate rich 3D behavior influenced by a wide range of angiogenic geometrical features and RBCs. Altogether, the findings present a new 3D picture of the hemodynamic microenvironment likely experienced by ECs during angiogenic network remodeling in vivo and suggest a conceptual foundation for considering the regulatory involvement of shear stress on EC biology.

## Materials and Methods

The approach employed in the current work involves integrating high-resolution images of angiogenic microvascular networks in the rat mesentery with high-fidelity RBC-resolved simulations in 3D. Output data from the simulations includes detailed information on the near-wall fluid dynamics, which were used to calculate 3D WSS and its spatial patterns. The sections below describe the methods employed for each of the respective components.

### RBC-Resolved Simulations

**Figure 1.**
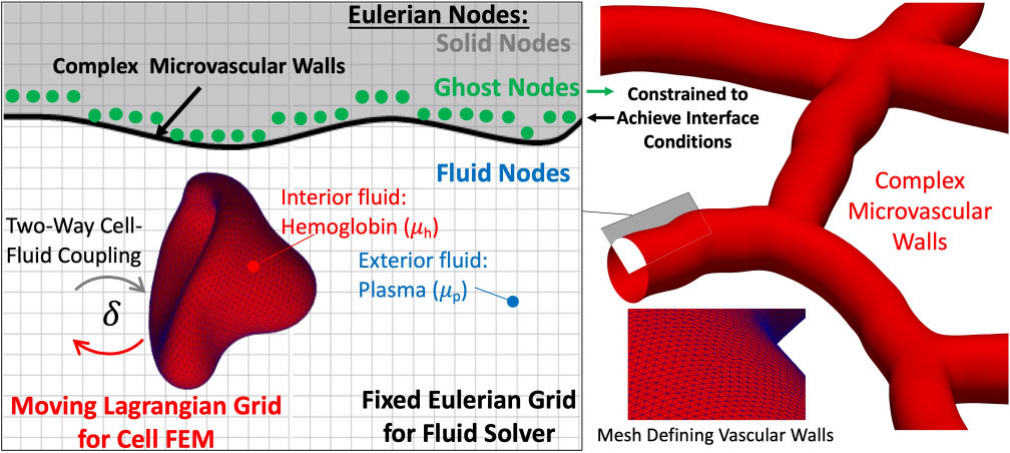
Overview of in silico method for modeling 3D RBC-resolved flows in complex geometries.

Three-dimensional computational fluid dynamics simulations are performed using an immersed boundary method-based approach for modeling biophysical flows in complex geometries.^72^ Salient details of the method are provided below, with a graphical overview provided in Figure 1. The method has been extensively validated against both theory and alternate numerical methods,^72^ as well as data from in vivo experiments.^8,69,73^ That this numerical approach can reproduce what has been generally observed in vivo was demonstrated in prior work. Here, we extend this to understand angiogenic WSS characteristics based on real image data.

Blood is modeled as a suspension of 3D RBCs flowing with plasma, and the cells deform as they flow through the complex 3D microvessels. Each RBC is represented by an infinitesimally thin elastic membrane enclosing a viscous fluid representing hemoglobin. Both plasma and hemoglobin are modeled as Newtonian fluids, with different dynamic viscosities; plasma properties are similar to water (1.2 cP), while the hemoglobin is modeled as being five times more viscous.^68^ Each RBC membrane is a surface defined by a Lagrangian mesh of 5120 triangular elements, and simulations each resolve approximately 1500 RBCs. The RBC resting shape is taken to be a biconcave discocyte with a major axis diameter of 7.8 µm, a surface area of 134.1 µm^2^, and a volume of 94.1 µm^3,68,74^ which correspond to human RBCs. Differences in major diameter between human and rat RBCs are approximately 0.8 µm,^75^ and reasoning behind the use of human RBCs is provided in the Limitations and Additional Considerations section. The governing fluid flow equations are the unsteady Stokes equations for a constant density (*ρ*) variable viscosity (*μ*) fluid $\rho \frac{{\partial {\boldsymbol{u}}}}{{\partial t}} = - \nabla P + \nabla \cdot [ {\mu ( {\nabla {\boldsymbol{u}} + \nabla {{\boldsymbol{u}}}^T} )} ] + {\boldsymbol{F}}$. Note that this considers a dynamic viscosity field that varies in time and space, or $\mu = \mu ( {{\boldsymbol{x}},t} )$. At a given time and instantaneous RBC configuration, the spatial locations outside RBCs have plasma viscosity, while inside RBCs have a viscosity five times higher. Mathematically we model this as $\mu ( {{\boldsymbol{x}},t} ) = {\mu }_p + ( {{\mu }_c - {\mu }_p} )I( {{\boldsymbol{x}},t} )$, where ${\mu }_p$ and ${\mu }_c$ are viscosities as denoted in Figure 1, and $I( {{\boldsymbol{x}},t} )$ is an indicator function used to track the separate fluids. Full details on the model and implementation can be found in prior works.^72,73^ The governing fluid flow equations are solved for the fluid velocity (***u***) and pressure (*P*) on a fixed, uniform Eulerian mesh with a projection method using a finite volume/spectral approach, where the body force term ***F*** incorporates stresses from the deformable cell model described below. Grid spacing for the fluid field is approximately 200 nm.

Complex vascular walls are modeled with a sharp-interface ghost node (GN) IBM, which decomposes the Eulerian computational domain into a fluid domain inside the vessels and a solid domain outside. The interface conditions at the walls are enforced when solving the fluid flow equations via constraints imposed at the Eulerian mesh points immediately outside the fluid domain (points termed as ghost nodes). Each GN has a corresponding image point (IP) in the fluid domain, which is the mirror image of the GN across the nearest location on the wall boundary, termed boundary intercept (BI). The velocity at the BI point is taken as the average of the GN and IP velocities, or ${{\boldsymbol{u}}}_{\mathrm{ BI}} = \frac{{{{\boldsymbol{u}}}_{\mathrm{ GN}} + {{\boldsymbol{u}}}_{\mathrm{ IP}}}}{2}$. Thus, to enforce a no-slip condition at the BI point, the velocity at the GN is written as ${{\boldsymbol{u}}}_{\mathrm{ GN}} = - {{\boldsymbol{u}}}_{\mathrm{ IP}}$ for ${{\boldsymbol{u}}}_{\mathrm{ BI}} = 0$. While the GN velocity is necessarily at an Eulerian mesh point, the IP is not. Thus, we represent the velocity at the IP based on the velocities at the 8 mesh points surrounding the IP using a trilinear interpolant of the form ${{\boldsymbol{u}}}_{\mathrm{ IP}} = \mathop \sum \limits_{i = 1}^8 {\beta }_i{{\boldsymbol{u}}}_i$, where ${\beta }_i$ are the interpolation weights.

The membrane stresses in response to deformation are calculated using the finite element method, with loop elements used as a subdivision surface for the force calculations.^76,77^ The surface force density ${{\boldsymbol{f}}}_e$ at each triangular element is determined by evaluating the surface gradient of the Cauchy tension tensor (${T}^{mn}$), and membrane (${\sigma }^{mn}$) and bending (${\nu }^{mn}$) stresses as ${{\boldsymbol{f}}}_e = {\nabla }_s( {{T}^{mn} + {\sigma }^{mn} + {\nu }^{mn}} )$. Force densities are transferred from elements to vertices using box-spline shape functions, and the vertex forces ${{\boldsymbol{f}}}_v$ are spread to the Eulerian mesh via ***F*** using a delta function in terms of Eulerian grid location ***x*** and membrane vertex location ***X***: ${\boldsymbol{F}} = \mathop \smallint \limits_S^{} {{\boldsymbol{f}}}_v\delta ( {{\boldsymbol{x}} - {\boldsymbol{X}}} )d{\boldsymbol{X}}$. This same delta function facilitates interpolation of the Eulerian velocity field to update the membrane vertex locations and deform the cell. To evaluate the Cauchy tension tensor an energy function developed for a RBC^78^ is used to determine the stress response to the strain as ${W}_s = \frac{{{G}_s}}{4}[ {( {I_1^2 + 2{I}_1 - 2{I}_2} ) + CI_2^2} ]$, where ${G}_s$ is the membrane shear elasticity, and ${I}_1$, ${I}_2$ are the strain invariants. The membrane can undergo significant deformation yet is nearly area-incompressible, in agreement with physiological behavior of RBCs.^79^ Resistance to surface area changes is enforced by the constant *C* in the previous equation for strain energy. A larger value of *C* results in increased area preservation but at the expense of numerical stability. Here, we have used *C* = 100, which we observe keeps the surface area changes to less than 0.1%. The Helfrich bending energy is used for the membrane and bending stresses, written as ${\sigma }^{mn} = \frac{{{k}_b}}{2}( {4{\kappa }^2{A}^{mn} - 8\kappa {B}^{mn}} )$ and ${\nu }^{mn} = \frac{{{k}_b}}{2}( {4\kappa {A}^{mn}} )$, respectively, where ${k}_b$ is the bending modulus, $\kappa $ is the mean curvature, and *A* and *B* are the metric and curvature tensors. Computations follow that described elsewhere.^80^

**Figure 2.**
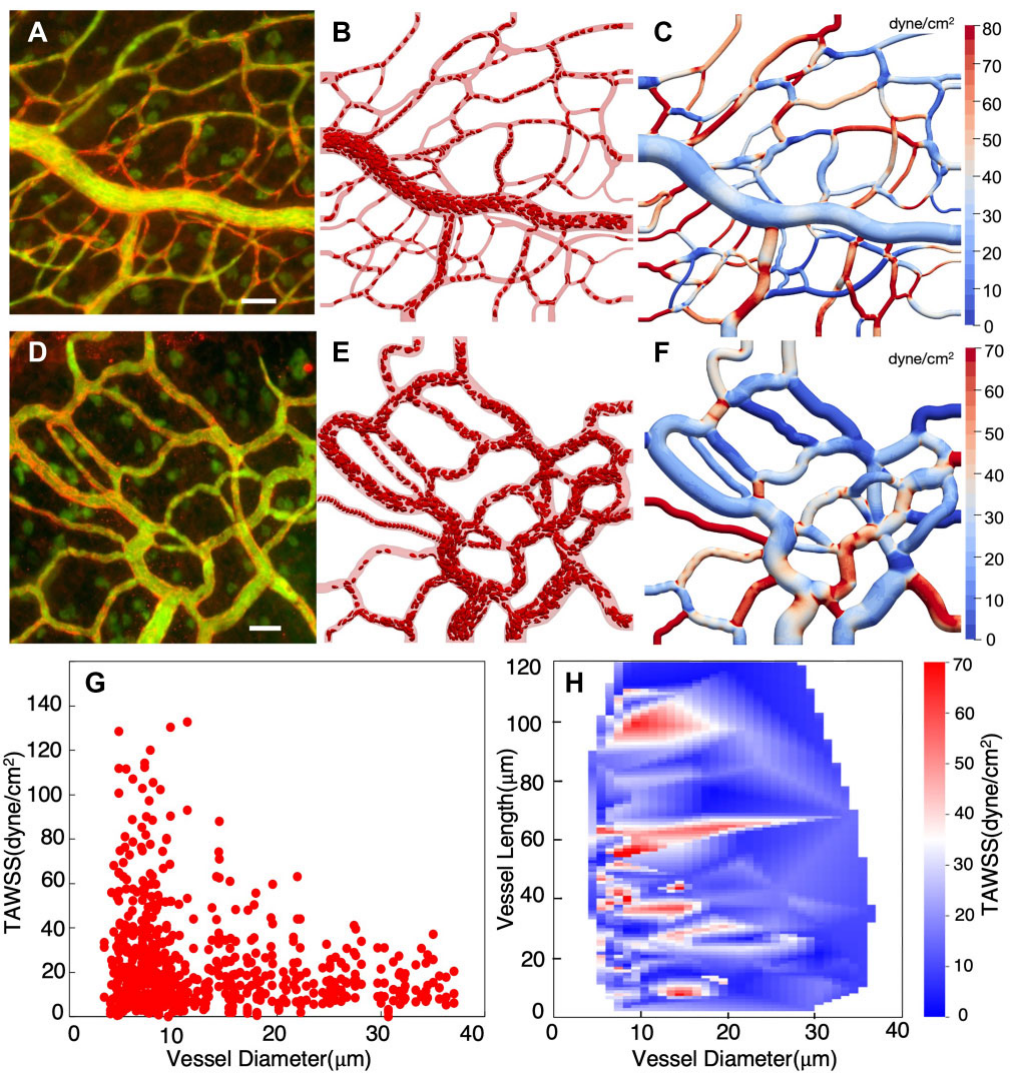
3D RBC-resolved simulations in angiogenic microvascular networks. (A) Image data for network 1, scale bar is 40 µm; (B) snapshot of RBC-resolved simulation; (C) surface contours of the time-averaged WSS, (D–F) same information but for network 2, (G) predicted time-averaged WSS (TAWSS) per vessel versus diameter, (H) heatmap of TAWSS per vessel as a function of vessel length and diameter.

Simulation snapshots shown in Figure 2 depict the entire domain of each modeled network, with vessels at the borders being either inlets or outlets. Flow rates are imposed at the inlets and outlets to achieve physiological effective shear rates generally in line with reported values in the literature for the mesenteric microcirculation.^81^ For each network, three different flow conditions are modeled, and denoted as baseline, 0.5x and 2x. Baseline shear rates at boundaries range from approximately 30 to 500 s^−1^, and are scaled for the other flow cases based on the denoted factor. Details are provided in the Supplementary Materials. At inlet vessels, hematocrit levels are maintained by adding RBCs from external, linked simulations involving RBCs flowing through straight tubes of the same diameter as the respective inlets. The inlet hematocrit values are prescribed generally based on vessel diameter following physiological values reported for the microcirculation,^81,82^ and range from approximately 12% to 36%. Details are provided in the Supplementary Materials on how RBC injection is handled at inlets, as well as the initial placement of RBCs throughout the networks. It is noted that for some inlet vessels the hematocrit slowly decreases over time, and this behavior is discussed in the Supplementary Materials.

### Calculation of WSS and WSSG

The high-resolution 3D velocity field enables calculation of the WSS and its gradient (WSSG) on the complex microvascular wall surfaces. To facilitate study of these quantities, we calculate the traction vector (***t***) at each vertex of the mesh defining the vascular walls. Specifically, ***t*** gives the force per area at the surface, and is related to the stress tensor ${\boldsymbol{\sigma }}$ by ${\boldsymbol{t}} = {{\bf n}} \cdot {\boldsymbol{\sigma }}$, where **n** is the unit normal vector at the surface. Following previous work,^8^ we use a local cylindrical coordinate system for the WSS calculation at each wall point due to the geometric complexity. The axial direction (${{\boldsymbol{e}}}_a$) points along the direction of the mean undisturbed flow near the wall, based on the idea that the mean flow moves in the axial direction. The radial direction (${{\boldsymbol{e}}}_r$) is in the inward-normal direction, and the circumferential component (${{\boldsymbol{e}}}_\theta $) is defined as ${{\boldsymbol{e}}}_\theta = {{\boldsymbol{e}}}_a \times {{\boldsymbol{e}}}_r$. This approach provides a consistent means of calculating WSS at all wall points and, furthermore, provides a convection that is similar in spirit to that used with standard Poiseuille flows in straight tubes. With this convention, the viscous components of the traction vector can be represented as ${t}_a = {\mu }_p\frac{{\partial {u}_a}}{{\partial r}}$, ${t}_\theta = {\mu }_p\frac{{\partial {u}_\theta }}{{\partial r}}$, and ${t}_r = 2{\mu }_p\frac{{\partial {u}_r}}{{\partial r}}$, where ${\mu }_p$ is the plasma viscosity since the calculation is at the vessel wall. While we observe the axial component to be many orders of magnitude greater than the other components, the WSS values reported here are taken to be the magnitude of the traction vector to include all data.

To calculate the WSS, the derivates in the equations for the traction components are numerically evaluated using second-order differencing inwards from the surface. The velocity components at each location in the stencil are first interpolated from the velocity field data, and then converted to the local coordinate system at the vertex. Thus, given a 3D velocity field determined from an RBC-resolved simulation, the resulting WSS at all surface points is calculated following this procedure. Subsequently, with the WSS determined its spatial gradients can be calculated. We calculate the WSSG components in both the axial and circumferential directions through surface interpolation of the WSS field and using second-order central differencing.

### Imaging of Angiogenic Microvascular Networks and Digital Reconstruction

Network images were obtained by re-imaging of angiogenic adult rat mesenteric tissues from a previous study.^83^ As described therein, angiogenesis was stimulated via i.p. injections of compound 48/80, a mast cell degranulator. This stimulation model was selected because it has previously been characterized to result in an increase in mesenteric microvascular network vascular area, increased capillary sprouting, and an increase in vascular length.^83–86^ The representative angiogenic network images for the current study were obtained from adult Wistar rat mesenteric tissues 10 d post stimulation.^86^ Our previous characterization of microvascular remodeling responses post stimulation in these tissues documented that vascular area per tissue is increased by day 10. Capillary sprouts per vascular area were increased by 3 d post the injection protocol and remained increased at day 10. At day 10, vascular length per vascular area was increased. Remodeled microvascular networks also displayed an increase in venule tortuosity and capillary plexus regions reflective of patterning changes.

Post stimulation, perfusion of fixable 40 kDa FITC-dextran identified vessel lumens. Vessel segments were additionally confirmed by labeling of blood ECs for PECAM. Mesenteric tissues were whole mounted and observed en face. The mesentery’s thinness (20–40 µm) enables imaging of intact networks at the vessel and cellular levels and eliminates the need for tissue sectioning. Microvascular networks exhibited increased segment density,^83^ which is characteristic of angiogenesis regardless of the stimulation method. Thus the 48/80 stimulated networks are sufficient to provide examples of angiogenic network regions. Two representative networks of high-density capillary regions were selected and captured using 10x (dry, NA = 0.3) objectives on an inverted microscope (Nikon Ti2) coupled with an ANDOR Zyla sCMOS camera.

For the present work, the angiogenic microvascular regions (Figure 2) were digitally reconstructed for 3D RBC-resolved simulations and analysis. PtcCreo^87^ was used to build the networks by first importing images on a plane, a reasonable approximation given the mesentery’s thinness as noted above, and extracting approximations for vessel centerline trajectories. Any out-of-plane vessels are identified through visual inspection, and centerlines modified accordingly. The 3D interconnected vessel structures are then created by sweeping circular cross-sections along the trajectories, with sizes adjusted locally in accordance with the images. The shapes of connection regions are similarly patterned to match the images. It is noted that the use of centerlines and locally circular lumen shapes is a modeling assumption due to images being 2D. Geometric details of the 3D network surfaces are then exported in .stp format and imported into the Gmsh software^88^ for surface triangulation. Such meshing results in highly resolved 3D discretized microvascular surfaces that comprise each network, with triangles having edge lengths of approximately 150 nm. The angiogenic network in Figure 2A–C has 9.6 million vertices and 19.2 million triangular faces, while that in Figure 2D–F has 7 million vertices and 14 million triangular faces. Geometries are stored in .stl format, which are used to represent the microvascular walls for the RBC-resolved simulations.

## Results

### Angiogenic WSS Patterns Exhibit Significant Heterogeneity from Vessel to Vessel

Surface contours of the time averaged WSS (TAWSS) predicted for each of the angiogenic microvascular networks are shown in Figure 2 from representative simulations. In addition to the TAWSS maps, Figure 2A–C and D–F provide snapshots of RBCs flowing through each respective network along with the image data from which the networks were reconstructed. Movies are also provided in the Supplementary Materials. Time-averages are performed over the entire simulation time for all cases, which is on the order of one second.

**Figure 3.**
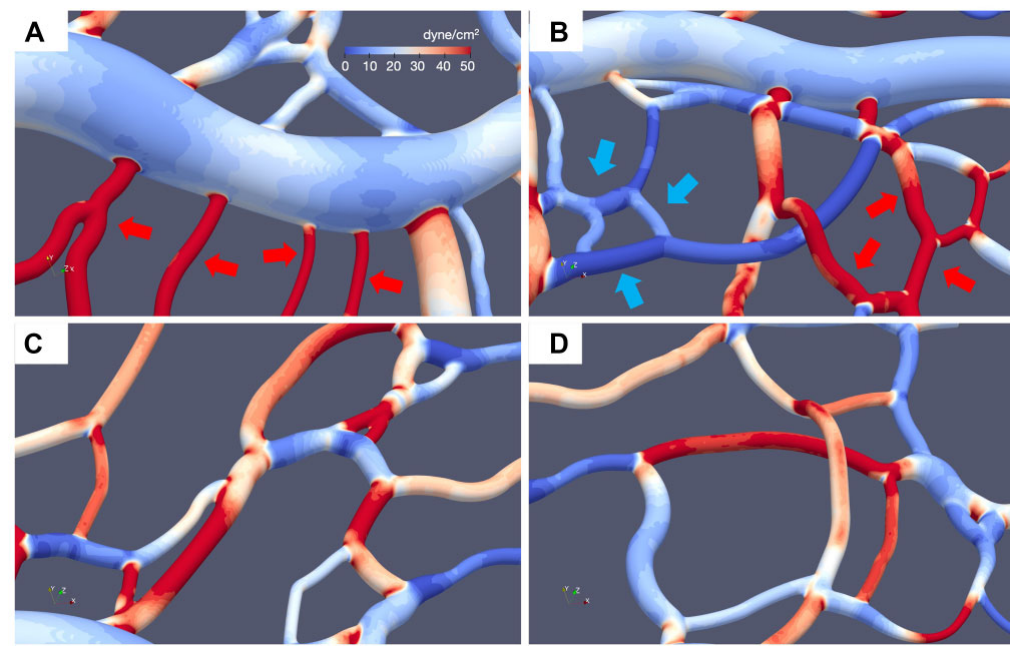
Vessel to vessel TAWSS heterogeneity. Representative images of 3D TAWSS surface contours predicted by simulations, illustrating the large degree of TAWSS variation from one vessel to another. (A) High TAWSS capillaries (red arrows) connecting to low TAWSS venule; (B) groups of low TAWSS vessels (blue arrows) connected to groups of high TAWSS vessels (red arrows); (C, D) high and low TAWSS vessels interconnected.

Significant TAWSS heterogeneity across each network is evident from the representative data shown in Figure 2C, F, where WSS contour values can be qualitatively observed to vary by almost two orders of magnitude between different vessels (see Figure 3 for zoomed-in perspectives of representative regions). To quantify this observation, the distribution of predicted TAWSS on a per-vessel basis (ie, spatially averaged over all points in a vessel) is given in Figure 2G for all simulations as a function of vessel diameter. From this figure, we observe the magnitude of the vessel-to-vessel TAWSS variability to be generally dependent on vessel diameter. Variations are most significant in the smallest capillary vessels (3–15 µm diameter), where values range from 0.01 dyne/cm^2^ to 130 dyne/cm^2^. With increasing diameter the vessel-to-vessel variability decreases, with TAWSS values approximately ranging from 0.01 dyne/cm^2^ to 40 dyne/cm^2^ in vessels greater than 25 µm in diameter. Additional perspective on heterogeneity on a per-vessel basis is provided by the heatmap in Figure 2H, which gives the TAWSS per vessel in terms of both vessel length and diameter. This data show that both high and low TAWSS vessels are scattered across all vessel lengths for diameters less than 20 µm. Similarly, for larger diameters the TAWSS per-vessel values (albeit lower in magnitude) are scattered across all vessel lengths without a clear pattern. These findings suggest that the TAWSS variability from vessel to vessel is not strongly influenced by vessel length in the reconstructed angiogenic networks, only the diameter (Figure 2G).

We observe that the enhanced TAWSS heterogeneity in small diameter vessels to be strongly influenced by the presence of RBCs. This is caused by increased confinement leading to increased RBC deformation and wall interactions, which, perhaps expectedly, leads to the high TAWSS values in small diameter vessels. This behavior, however, also contributes to low TAWSS in such vessels. The simulations predict per-vessel TAWSS values to occur in direct proportion to predicted vessel shear rates (see Supplementary Material), and in a few vessels there are negligible flows, which naturally leads to negligible TAWSS values. Some of these low-flow vessels have diameters 3 µm or less, which through physical occlusion effectively prevent the passage of RBCs. Through network structure, cells are routed to other adjacent vessels, which in many cases incur relatively high TAWSS. Interestingly, we also observe small diameter vessels devoid of RBCs can have TAWSS values upwards of 40 dyne/cm^2^ (see Supplementary Material and Discussion). Collectively, these behaviors are only captured by explicitly resolving the RBCs. We observe these patterns of vessels with low TAWSS adjacent to vessels with high TAWSS to occur for either single vessels or groups of vessels. Representative examples are provided in Figure 3.

### Complex 3D Spatial Variations Occur at Sub-EC Length Scales

**Figure 4.**
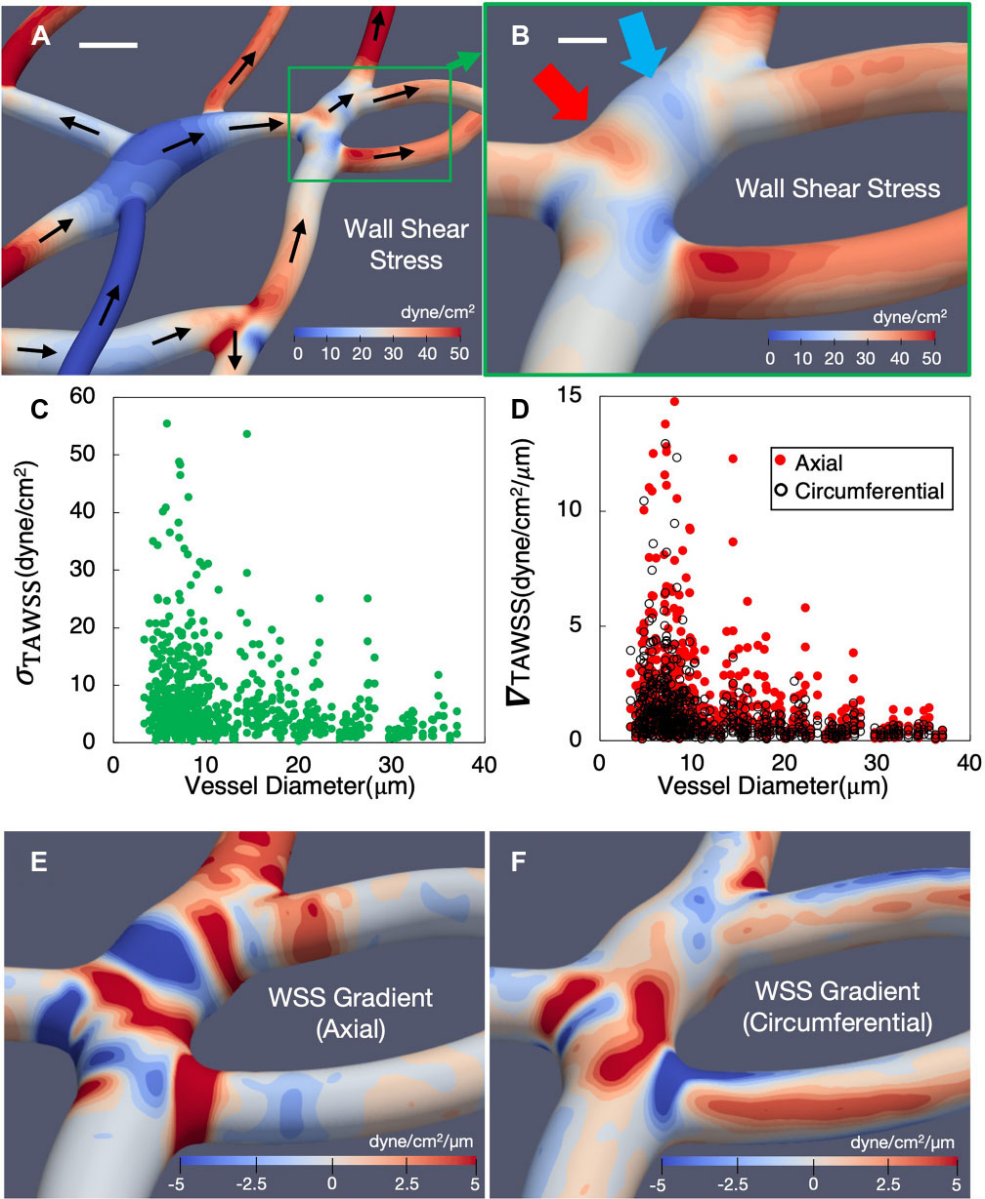
Angiogenic TAWSS spatial variations and gradients in 3D. (A) TAWSS contours for a representative angiogenic network region, scale bar is 10 µm, and arrows give the flow direction; (B) zoomed in view of a subregion from (A), detailing the complex TAWSS spatial patterns and significant variations, arrows point to representative hot and cold spots, scale bar is 5 µm; (C) standard deviation ($\sigma )$ of TAWSS versus vessel diameter, where each data point gives $\sigma $ for all surface points in a vessel; (D) spatial gradients of the TAWSS components (${\nabla }_{ax},{\nabla }_{circ}$) versus vessel diameter. Each data point gives the respective $\nabla $ component averaged over all surface points in a vessel. (E, F) Contours of the TAWSS spatial gradients (E, axial direction; F, circumferential direction) corresponding to the TAWSS patterns in (B).

Close inspection of the TAWSS surface contours in Figures 2 and 3 reveals complex 3D spatial patterns underlying the per-vessel TAWSS values. Representative examples of these patterns are provided in Figure 4 for one of the angiogenic network regions.

As evident from the representative behavior depicted in Figure 4A, TAWSS variations are observed on surfaces within individual vessels. Namely, both the magnitudes of the localized TAWSS values experienced at surface points as well as the degree of spatial variation among the vessels shown are highly heterogeneous. The 3D surface contours in Figure 4B provide a zoomed-in perspective illustrating characteristic behavior we observe, namely the formation of TAWSS hot and cold spots within vessels and sharp changes over relatively small distances. For the example vessel in Figure 4B, based on the TAWSS spatial patterns and the scale bar shown, significant TAWSS variations occur over distances that are *O*(µm), which are shorter distances than typical size of ECs that line vessel walls.

To quantify the magnitude of localized 3D spatial variations within vessels, we first compute the standard deviation (*σ*) of the TAWSS among all surface points in each vessel. Data shown in Figure 4E provides the predicted distribution of σ versus vessel diameter for all vessels. In the smallest capillaries σ values can reach as high as 60 dyne/cm^2^, while negligible values are observed as well in a few vessels. This date indicates that within vessels of similar diameter the degree of spatial variation in TAWSS can range over an order of magnitude. Spatial variations within vessels of larger diameter among the data points in Figure 4E show σ values reaching approximately 20 dyne/cm^2^, with a lower degree of vessel-to-vessel variation as compared to the smaller diameter vessels.

The structure to the TAWSS spatial patterns in Figure 4B qualitatively exhibits a directional preference, meaning sharp changes can occur in specific directions with respect to vessel orientation and flow direction. For example, the hot and cold spots associated with the identifying arrows in Figure 4B occur in sequence along the mean flow direction (see Figure 4A for flow direction), where a TAWSS hot spot is followed by a cold spot. This indicates that a dominant spatial change to the TAWSS occurs here in the direction of the mean flow or the axial direction. For this example, this occurs due to a temporary increase in vessel area based on the local morphology, which causes the observed spatial patterns exhibiting a negative TAWSS gradient in the axial direction (ie, a region of relatively high TAWSS transitioning to a region of low TAWSS). From the high-resolution simulation output we can mathematically quantify such behavior by computing the gradient of the TAWSS field in the axial direction (TAWSSG_axial_). Contours of this quantity are shown in Figure 4E calculated from the TAWSS field shown in Figure 4B. Figure 4D provides TAWSSG_axial_ averaged over all points in each vessel versus vessel diameter. Here, the absolute values are plotted to generally reflect the magnitude of the predicted spatial variations.

To add further perspective to the spatial variations quantified as spatial gradients, we also compute the TAWSS gradient in the circumferential direction (TAWSSG_circ_). Contours of this quantity are shown in Figure 4F, which augments the three-dimensional nature of the observed TAWSS spatial variations in the above example. Namely, the TAWSS does not simply decrease uniformly as the vessel cross-sectional area locally increases, but rather changes in a much more complex and directionally biased manner. Pronounced TAWSS axial gradients also occur within regions where vessels connect to one another, as in the lower-most horizontal vessel in Figure 4E. For this example, as the flow enters the smaller diameter vessel the fluid accelerates resulting in increased wall shear rates and increased TAWSS in the axial direction. As with the previous example, however, this spatial variation is much more complex than a simple increase in value. Instead, TAWSS values increase and spatial patterns change. This is quantified in Figure 4E, F by the regions of pronounced TAWSSG axial and circumferential components.

**Figure 5.**
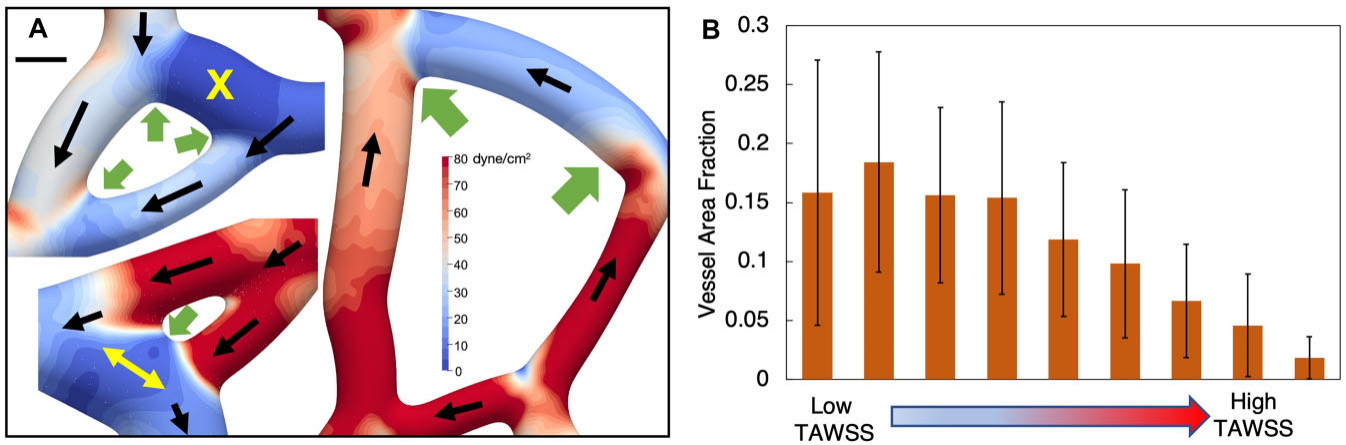
Spatial patterns around microvascular loops and average TAWSS distribution behavior in vessels. (A) Representative examples of microvascular loop structures and resulting TAWSS spatial patterns. Black arrows give the flow direction, yellow arrows and the X symbol denote a low flow multi-directional vessel and a no-flow vessel, respectively. Green arrows point to transition regions between connected vessels with significant TAWSS variations over short distances. Scale bar is 6 µm. (B) Histogram quantifying tendency of vessels to experience low or high TAWSS, on a per-area basis. Data are averaged over all vessels.

Characteristic angiogenic network structures are microvascular “loops,” where three or more vessels are connected to one another in an arrangement resembling a loop structure. Representative examples are provided in Figure 5A. From our observation of large TAWSS spatial changes at locations where vessels connect to one another, we also analyzed behavior in these loop structures. A hemodynamic behavior we observe that affects the TAWSS is at least one of the vessels comprising a loop typically has negligible flow of RBCs. For the angiogenic networks considered in this work, we identified 25 microvascular loop structures and among them 17 exhibited this behavior (see Supplementary Material). Due to such flow discrepancies amongst connected vessels, our simulations reveal that the resulting TAWSS patterns can have orders-of-magnitude spatial variations over very small distances. Examples are shown in Figure 5A, where for these loop structures one of the connected branches is a low-flow (or negligible flow branch). Interestingly, we also report low flow branches in which the flow direction varies over time, an example of which is given in Figure 5A and denoted by the yellow arrows. This occurs here just downstream of a bifurcation, in a cross-vessel where we observe RBCs to flow very slowly and in either direction depending on the local time-dependent configuration of flowing RBCs and their interactions (movie provided in the Supplementary Material).

Given the significant degree of TAWSS spatial variations within vessels, we also analyzed the tendency of individual vessel surfaces to experience high or low TAWSS values. To achieve this, we considered TAWSS values across all surface points for a vessel, and first binned the TAWSS across the range of values experienced for that vessel. Bin values for each vessel span the full range for the vessel from low to high TAWSS and are relative to the minimum and maximum TAWSS values for each vessel. Values are distributed over nine bins for each vessel, so that the lowest and highest bins correspond to the low and high TAWSS values for the vessel. Then, for each bin, we determined the corresponding vessel surface area fraction to provide a histogram of TAWSS behavior on a per-area basis for each vessel. These data are provided in Figure 5B, which give the average over all vessels. Interestingly, we observe that on average, the area fraction of a vessel experience high TAWSS tends to be very small, whereas areas experiencing low TAWSS tend to be large.

### RBCs Alter TAWSS Behavior in Angiogenic Networks

**Figure 6.**
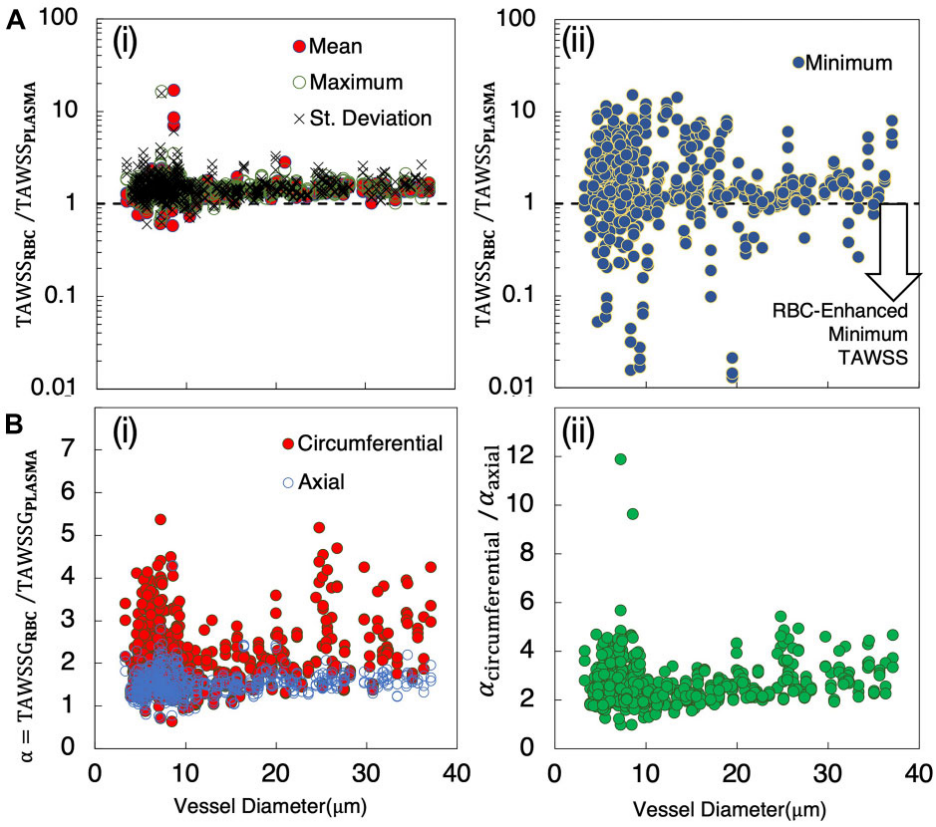
Alteration of TAWSS behavior in angiogenic networks due to RBCs. (A) Ratio of TAWSS per vessel from simulations with and without RBCs (${\mathrm{TAWS}}{{\mathrm{S}}}_{{\mathrm{RBC}}}/{\mathrm{TAWS}}{{\mathrm{S}}}_{{\mathrm{plasma}}})$ versus vessel diameter. (i) Ratio based on mean, maximum, and standard deviation of TAWSS per vessel, (ii) ratio based on minimum TAWSS per vessel, with values below 1.0 indicating vessels where the minimum TAWSS decreases due to RBCs; (B) TAWSSG per vessel with and without RBCs, versus vessel diameter. (i) Ratio ${\mathrm{\alpha }} = {\mathrm{TAWSS}}{{\mathrm{G}}}_{{\mathrm{RBC}}}/{\mathrm{TAWSS}}{{\mathrm{G}}}_{{\mathrm{plasma}}}$ for both circumferential and axial components, (ii) ratio of ${{\mathrm{\alpha }}}_{{\mathrm{circ}}}/{{\mathrm{\alpha }}}_{{\mathrm{axial}}}$ versus diameter.

**Figure 7.**
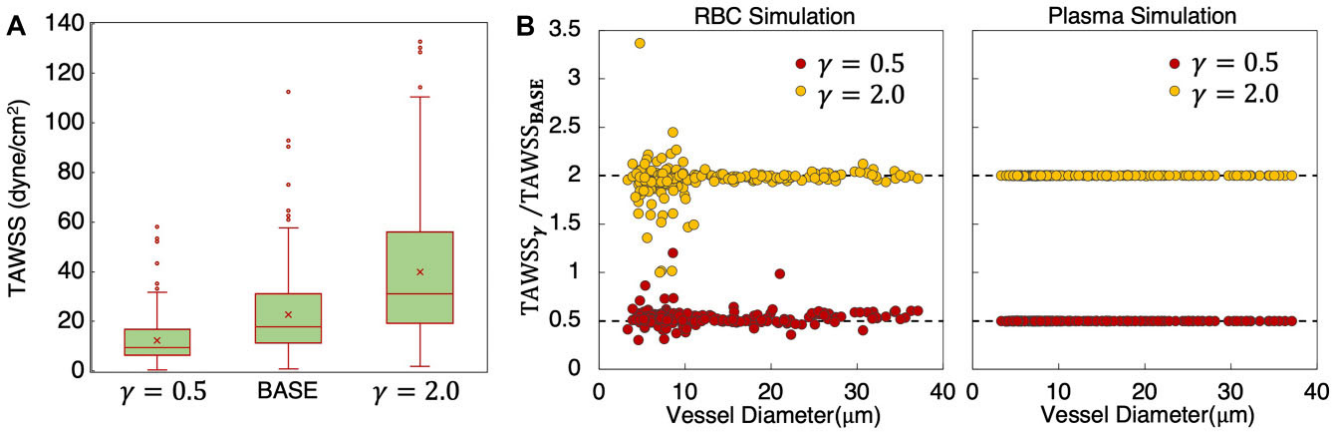
Influence of boundary conditions on TAWSS and influence RBCs. (A) Distribution of TAWSS values across networks for the baseline flow conditions at network boundaries (BASE, or *γ* = 1.0), as well as values for flows modified from the base by factors of 0.5 and 2.0 (*γ* = 0.5,2.0). (B) Ratio of TAWSS per vessel for each *γ* to the baseline conditions versus diameter. Left- and right-most plots give data from simulations with and without RBCs, respectively.

The presence of RBCs expectedly alters the TAWSS over that experienced based on pure plasma flow alone. We quantify this behavior by comparing the per-vessel TAWSS from the RBC-resolved simulations to the per-vessel TAWSS from simulations in the angiogenic networks without any RBCs (ie, only plasma). Differences manifesting in various aspects of the TAWSS character are quantified in Figures 6 and 7.

Comparative differences between TAWSS with and without RBCs are shown in Figure 6A. For each vessel, we compute the mean, maximum, minimum, and standard deviation of the TAWSS among all surface points and plot the ratio of these values versus vessel diameter for simulations with and without RBCs. Quantities are separately plotted in Figures 6A-i and A-ii, in order to isolate differences in behavior we observe. That is, in Figure 6A-i, the mean, maximum, and standard deviation ratios are plotted, and these quantities all exhibit similar behavior; values have similar magnitudes with respect to diameter, and are greater than unity for most vessels (ie, RBCs increase the quantity being considered). Specific values are generally between 1.5 and 3 for most vessels, while in some vessels values are less than unity. Reasons for the later are discussed in a subsequent section on local spatial variations, but in general occur due to vessels with enhanced cold spots caused by RBCs and/or vessels with very short length and complex morphology. The behavior in Figure 6A-i contrasts that of the ratio for minimum TAWSS, which is shown in Figure 6A-ii. While many values are indeed greater than unity, interestingly many vessels also have values less than unity. To best illustrate this, values are plotted in Figure 6A on a log scale. As indicated in the figure, data points that fall below the 1.0 line represent vessels in which the presence of RBCs has reduced the minimum value of TAWSS experienced in the vessel or enhanced the low TAWSS character.

The influence of RBCs on the TAWSS will naturally lead to influences on the TAWSSG. To quantify this influence, we plot the ratio of TAWSSG with and without RBCs in Figure 6B. Here, we denote this ratio as $\alpha = {\mathrm{TAWSS}}{{\mathrm{G}}}_{{\mathrm{RBC}}}/{\mathrm{TAWSS}}{{\mathrm{G}}}_{{\mathrm{plasma}}}$ to facilitate comparing the roles of the directional components via Figure 6B-i and B-ii. The $\alpha $ values for the axial and circumferential components are plotted in Figure 6B-i versus vessel diameter. As shown, the RBCs generally increase the TAWSSG across all vessel diameters, will values increasing by as much as five times compared to the plasma simulations. An interesting result is the increased $\alpha $ values for the circumferential component as compared to the axial component. That is, the presence of RBCs significantly enhances the TAWSSG circumferential component compared to the axial component. Recent work reported similar behavior,^8^ which we also show here for the angiogenic networks considered. To quantify the magnitude of enhancement to the TAWSSG circumferential component compared to the axial component, we plot the ratio of these $\alpha $ components in Figure 6B-ii. This generally shows that the enhancement ranges from 2 to 5 times across the range of vessel diameters.

For the angiogenic networks considered, simulations were performed over a range of driving flow rates (or, shear rates) at network boundaries, to investigate dependence of results on imposed boundary conditions. This is especially due to the RBC-resolved flows here, and the potential for a non-linear relation between the perfusion distribution across the networks and rate of flow increase at the boundaries due to the presence of the deformable RBCs. To investigate this relation, we consider data from our baseline simulation (see Materials and Methods) and compare against data from simulations where flows at all boundaries have been modified by factors of 0.5 and 2.0 (referred to here as *γ* = 0.5 and *γ* = 2.0). In Figure 7A, we first show the influence on the distribution of TAWSS values across the networks. As can be seen, the mean values roughly increase in proportion to *γ*. The TAWSS variability across the networks also increases with increasing *γ*. To better understand the TAWSS values that cause this behavior, in Figure 7B, we plot the ratio of TAWSS at each *γ* to the baseline case. Interestingly, for vessels with diameters less than 10 µm, the TAWSS per vessel does not scale in proportion to *γ* as would occur in the absence of RBCs (see right-most plot in Figure 7B for behavior from the plasma-only simulations). Instead, we observe highly scattered behavior, which emphasizes the role played by RBCs in influencing the perfusion distribution and resulting TAWSS behaviors.

### RBCs Enhance Both Hot and Cold Spots

TAWSS hot and cold spots, defined as localized and concentrated regions of high or low TAWSS values, are predicted in most vessels throughout the networks. The high and low values that define spots are relative quantities that depend on local TAWSS magnitudes, which we qualitatively identify by adjusting local contour values to the TAWSS range experienced in the field of view for network sections. While specific TAWSS values defining a hot or cold spot will necessarily vary, local variations by roughly 10 dyne/cm^2^/µm or greater tend to form spots that can be visually identified.

**Figure 8.**
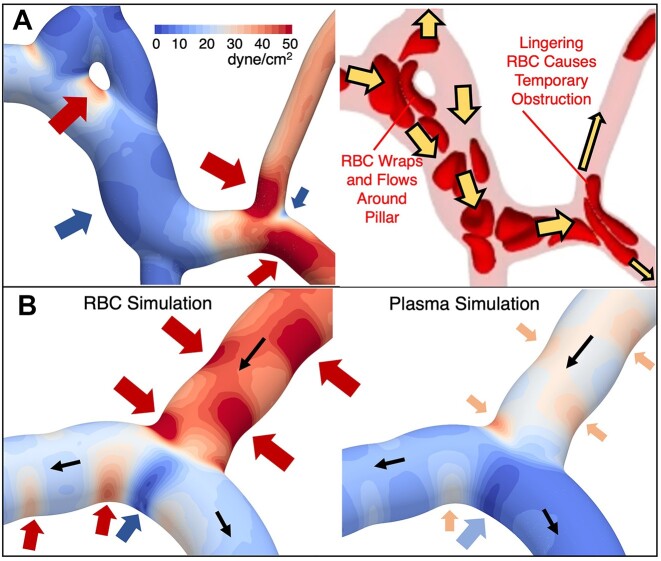
TAWSS hot and cold spots and enhancement by RBCs. (A) TAWSS field for a representative region, with specific hot and cold spots discussed in the main text identified by the red and blue arrows, respectively. Also shown is a snapshot of RBCs, depicting representative behavior that gives rise to the noted hot and cold spots. (B) Hot and cold spots within a representative bifurcation from simulations with and without RBCs The blue arrow points to an enhanced cold spot, which has a lower average value and is more focused with RBCs than without. Red arrows identify hot spots due to surface curvature changes, with hot spot intensity enhanced by RBCs.

Hot or cold spot formation is necessarily connected to highly localized TAWSS spatial gradients, which conceptually follows the previous discussion associated with Figure 4B. The enhancement of TAWSS hot spots by RBCs is expected, and we find this behavior can manifest by increasing both the magnitude and area of the hot spot. For TAWSS cold spots, enhancement interestingly causes the minimum TAWSS to be even smaller and the region much more focused. Such behavior underlies the behavior quantified in Figure 6B-ii. Representative examples of hot and cold spot patterns and influence by RBCs are provided in Figure 8, which complement the patterns in Figures 4 and 5.

A primary location where RBCs enhance TAWSS hot spots is within regions where vessels connect to one another. Depending on the local geometry, however, hot spot enhancements can manifest differently. For the region shown in Figure 8A, the RBCs enter from the upper left and wrap around the pillar-like obstruction as they flow downstream. For this case, the near-wall motion of the RBCs causes a TAWSS hot spot on the side of the pillar as noted by the red arrow in the figure, which is enhanced compared to behavior for plasma-only simulations by nearly five times (see Supplementary Material). Such enhancement is intuitive given the expected increase in near-wall velocity gradient caused by the presence of RBCs. In contrast, other behaviors shown in Figure 8A demonstrates hot spot formation in non-intuitive locations. This can be seen in the lower-right region of the figure, where due to increased confinement, an RBC will tend to linger on the bifurcation apex and temporarily obstruct the flow. Other RBCs and plasma are subsequently re-routed locally around the blockage and cause increased velocity gradients through these temporarily reduced flow areas. This leads to TAWSS hot spots on the outside wall, as noted by the red arrows. The formation of these hot spots is due to the discrete, particulate nature of the 3D RBCs, and would not occur with a continuum blood model. Along these lines, we note that such behavior is not enhanced by RBCs as is observed for other types of hot spots, but rather is caused by RBCs.

This bifurcation apex in Figure 8A marks the location of a TAWSS cold spot, as indicated by the blue arrow in the figure. Such a cold spot is also observed in the plasma-only simulations and occurs in both cases because it is a flow stagnation point. The impact of RBCs lingering on the apex interestingly reduces the TAWSS magnitude compared to the plasma-only simulations, but also causes the area of the cold spot to be reduced. Such cold spot enhancement, which manifests in a more focused region, is given in Figure 8B, where TAWSS contours are shown for both RBC and plasma-only simulations. The reduced cold spot area is generally caused by the narrowing of the cold spot due to adjacent hot spots enhanced by the near-wall movement of RBCs flowing through the bifurcation and moving downstream. This adjacent hot spot enhancement is denoted in Figure 8B by the red arrows in the bifurcation on either side of the cold spot, along with comparison to the contours from the plasma simulation. The existence of the hot spot immediately to the left of this cold spot in the bifurcation is caused by changes to the local surface curvature of the vessel wall, which is another primary means by which we observe hot and cold spots to form. At this location, the vessel bends back and forth in the downstream flow direction, resulting in a region of high surface curvature at the location of the hot spot, followed by a region of low surface curvature where the TAWSS is reduced. Such variations in TAWSS following the vessel curvature generally occur due to the shifted velocity profile for creeping flows in curved vessels; the velocity profile in a curved tube at low Reynolds numbers will be skewed toward the side of the vessel with the higher curvature resulting in larger near-wall velocity gradients.^8,69^ For the present work in angiogenic vessels, this manifests in patterns such as that shown in Figure 8B within the vessel feeding the bifurcation where hot spots shift back and forth between sides following changes to the surface curvature. This behavior is also observed for the corresponding TAWSS contours for plasma simulations, demonstrating that this phenomenon is caused by the geometry but enhanced by the RBCs. This can also result in TAWSS cold spots such as that denoted by the blue arrow on the left-most side of Figure 8A.

### Time-Dependent WSS Fluctuations Follow Timescales of RBC “Footprints”

**Figure 9.**
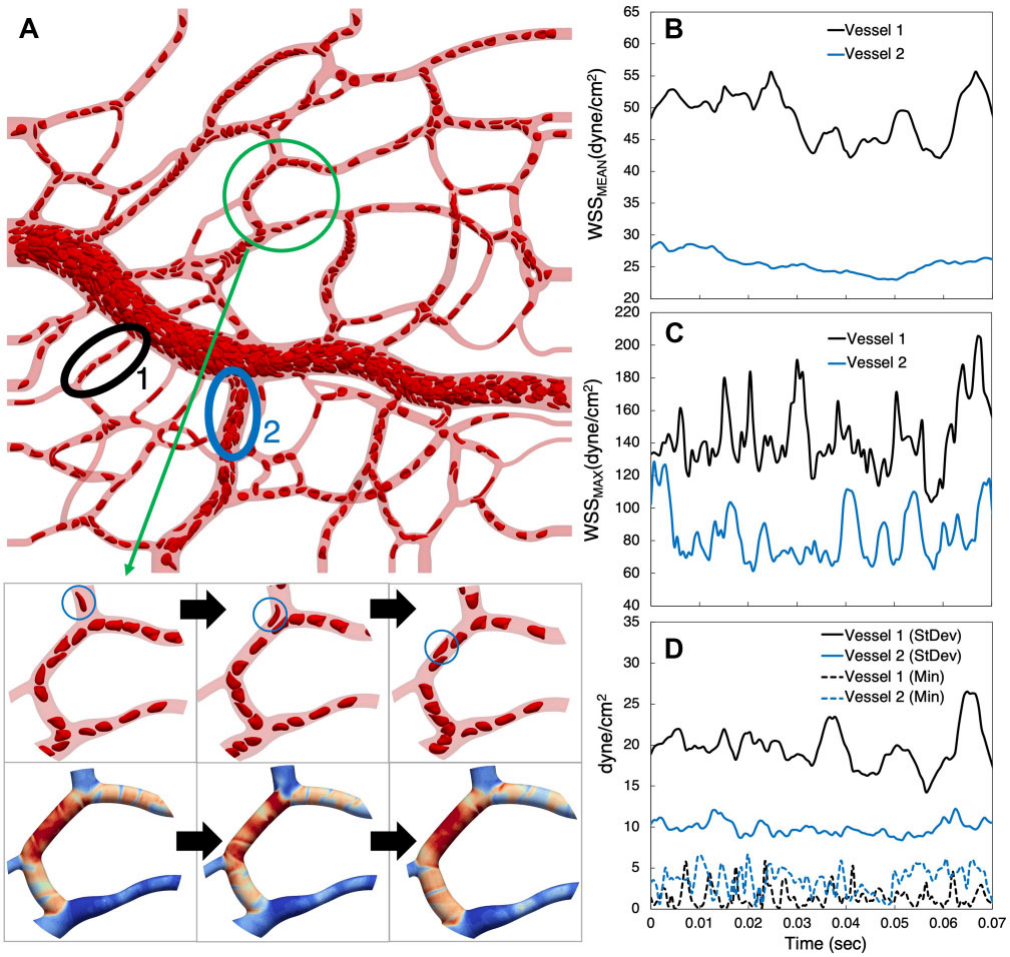
Time-dependent WSS behavior. (A) Snapshot of RBCs at an instant in time. Inset frames track the motion of the circled RBC as it moves through the vessel, with corresponding instantaneous WSS patterns. (B–D) Time signals for WSS statistics from two representative vessels circled in (A). (B) Mean vessel WSS, (C) maximum vessel WSS, and (D) standard deviation and minimum vessel WSS.

The presence of individual deformable RBCs flowing through small microvessels creates WSS spatial patterns that move with the RBCs. In the present work, this time-dependent behavior occurs entirely because of the RBCs, as the boundary conditions are constant. This enables isolation of the RBC contribution to the time-dependent WSS behavior, which occurs on different timescales than the cardiac cycle. For example, an RBC flowing through a microvessel at a velocity of 0.5 mm/sec will traverse a distance of 10 µm in 0.02 s. From the simulation data, we observe the resulting RBC “footprint” on the WSS, as termed in previous work in idealized vessels,^89^ to approximately follow such timescales resulting in WSS fluctuations. Figure 9 provides representative examples that depict and quantify aspects of the time dependent WSS predicted by the 3D simulations.

To illustrate the relationship between instantaneous WSS spatial patterns and the movement of individual RBCs, the inset to Figure 9A depicts WSS contours at three sequential time points along with the corresponding RBC positions. At each time point, the WSS contours exhibit spatial variations, and these can be observed to change following the RBC motion. This observation augments the concept of WSS heterogeneity by revealing how the sub-EC scale spatial variations are constantly in flux. To quantify the degree of WSS variations in both space and time, Figure 9B–D provide example time signals for different WSS spatial statistics from two separate vessels identified in Figure 9A. To reveal behavior resulting from different hemodynamics expected in angiogenic networks, we focus on (1) a small capillary where RBCs flow in single file and (2) a larger diameter vessel where RBCs flow in multifile fashion. The figures focus on behavior over a time interval of 0.07 s in order to highlight the timescale of the fluctuations in quantities.

First, shown in Figure 9B is the time signal for the mean WSS (ie, WSS spatially averaged over all points in the vessel at each time instant). As can be seen, the small capillary fluctuates roughly between 45 and 55 dyne/cm^2^, with an oscillation timescale of approximately 0.021 s. We calculate this timescale as the inverse of the dominant frequency resulting from a fast Fourier transform of the time-series data. Temporal variations for the larger vessel are smaller, with values ranging from approximately 25 to 30 dyne/cm^2^. Next, in Figure 9C, we plot the maximum vessel WSS versus time where the small capillary fluctuates between 120 and 190 dyne/cm^2^ with an oscillation timescale of approximately 0.013 s, and the larger vessel from 60 to 130 dyne/cm^2^ with a similar timescale. The differences between the magnitude of these values and their fluctuations are significantly enhanced compared to the mean fluctuations, providing a measure quantifying the degree to which the spatial variations over the vessels change with time. To quantify the time-evolving spatial variations further and more directly, we plot the WSS standard deviation over the two vessels versus time in Figure 9D. The time signals for the standard deviation show values ranging from 15 to 26 dyne/cm^2^ for the small capillary with an oscillation timescale of approximately 0.016 s, and 7 to 12 dyne/cm^2^ for the larger vessel. To fully quantify oscillation timescales for the WSS metrics considered (ie, mean, maximum, minimum, and standard deviation), data for all vessels is provided in the Supplementary Material. Similar to other quantities in previous sections, the oscillation timescales show significant heterogeneity in smaller capillaries, which decreases with increasing diameter.

**Figure 10.**
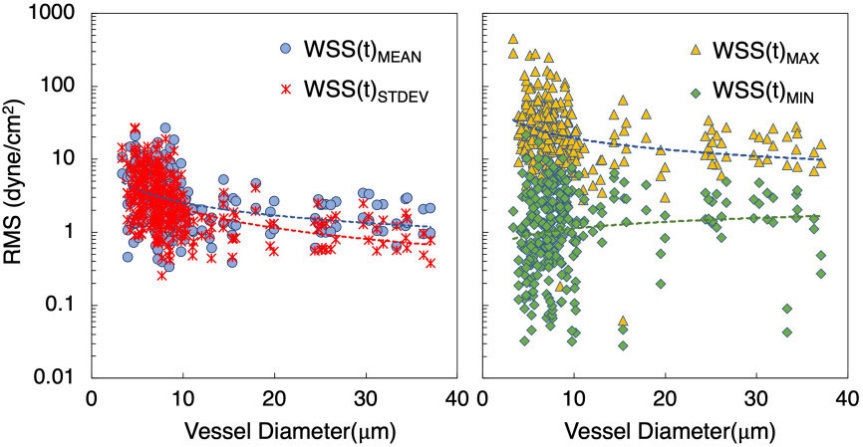
Time-dependent WSS fluctuations within vessels. RMS values of the time dependent fluctuations of WSS statistics versus vessel diameter. The left-most plot gives RMS values for the mean and standard deviation WSS time signals for each vessel, while the right-most plot gives RMS values for the maximum and minimum WSS signals. Best-fit lines corresponding to each data set are also provided based on a power-law regression.

Having established and quantified WSS fluctuations in representative angiogenic vessels, we also quantified time-dependent fluctuations predicted across all network simulations. To facilitate this, we calculated for each vessel the root mean squared (RMS) of the fluctuations for WSS mean, maximum, minimum, and standard deviation time signals. The data are provided in Figure 10 as a function of vessel diameter. As with our previous observations on TAWSS, the degree of heterogeneity can be seen to generally increase with decreasing diameter. For the WSS mean and standard deviation trends, RMS values are very similar, and generally range from 0.5 to 30 dyne/cm^2^. In contrast, RMS values for the maximum WSS values in each vessel range from 10 to 500 dyne/cm^2^, indicating significant temporal fluctuations can occur in some capillaries. A similar range for RMS of the minimum WSS in each vessel is observed, except magnitudes from 0.01 to 10 dyne/cm^2^. Similarity between the mean and standard deviation RMS values occurs because the WSS spatial distributions tend to move with the RBCs as mentioned, and these quantities represent aggregate behavior over whole vessels. This results in more gradual change with time relative to the RMS values of the WSS extremum, which are naturally much more volatile.

## Discussion

The findings in this study reveal high-resolution 3D WSS patterns in angiogenic microvascular networks based on real image data. Specifically, the results provide novel quantification of 3D WSS magnitudes, spatial, and temporal characteristics based on 3D RBC-resolved simulations in real angiogenic network structures. While 3D WSS variations over sub-EC length scales have been previously suggested for microvascular networks by prior works, for example,^7,8,90^ these previous studies involved either idealized or unstimulated networks. The qualitative depictions and quantitative analyses presented here thus provide insights to the potential shear stress microenvironment likely experienced by ECs in real angiogenic scenarios. Consequently, the findings lay the groundwork for furthering mechanistic understanding of EC behavior in remodeled networks and emphasize the need to consider shear stress as non-uniform stimuli that varies over space and time in a highly localized manner.

An underlying goal of this work is to provide representative insights and quantifications of the types of WSS patterns that can occur when 3D RBCs and angiogenic network morphologies are resolved. With this resolution, and the significant degree of WSS spatial variability shown here to result, questions then arise as to how such WSS complexity could possibly guide angiogenesis. While the current understanding is based on more coarse-grained representation, the current findings provoke re-consideration of the roles played by WSS in affecting angiogenic behavior. As models become more advanced, the increased granularity provides an opportunity to build new mechanistic models to understand angiogenesis in a more localized and idiosyncratic fashion, even on a patient-specific basis.

The prediction of vessel-to-vessel TAWSS heterogeneity, and the significant range of values among different vessels of similar caliber, provides baseline behavior to establish context for subsequent analysis. We have shown that on a per-vessel basis, TAWSS values can range anywhere from negligible to 130 dyne/cm^2^. *In vitro* works investigating EC responses to applied forces have shown values in this range to have angiogenic significance. For example, works by dela Paz et al.^3^ and Gloe et al.^4^ demonstrated that when ECs were exposed to shear stresses ranging from 10 to 30 dyne/cm^2^ the secretion of pro-angiogenic growth factors (ie, bFGF) and the upregulation of growth factor receptors (ie, VEGF-R2) were promoted. Similar stress values were shown to promote EC sprouting,^1^ while others have shown EC responses triggered by lower stress values, for example, 3–10 dyne/cm^2^,^58,91^ or higher stress values upwards of 100 dyne/cm^2^.^59^ While such works have provided important data for inferring the force magnitudes experienced by ECs during angiogenesis, in vitro conditions are known to be very different from in vivo environments.^92^ The direct calculations of TAWSS magnitudes reported here per vessel from real angiogenic networks are generally in agreement with what can be inferred based on the in vitro works. However, our findings importantly show and quantify how representing a vessel with one WSS value neglects the rich 3D spatial variations that are likely observed during angiogenesis in vivo.

Underlying the TAWSS vessel-to-vessel variability is heterogeneity in blood flow distributions throughout the networks. Notably, we observe behavior similar to recent work that reported heterogeneity of RBC perfusion among connected vessels during microvascular remodeling.^93^ This alludes to the concept of a preferential path taken by RBCs through networks, resulting in regions where some vessels are always filled with RBCs and other vessels are always empty. The impact of this behavior on TAWSS naturally contributes to the TAWSS per-vessel heterogeneity, and expectedly in our simulations we observe vessels with negligible TAWSS values to be generally devoid of RBCs. Interestingly, however, the opposite is not always true; vessels generally devoid of RBCs do not always have negligible TAWSS values. In vessels with diameters generally less than 5 µm, we observe that RBCs can, in some cases, be routed to other connected vessels due to more favorable hydrodynamic resistance. Yet, the RBC-free vessel is non-negligibly perfused by plasma. For such cases, we observe that TAWSS values can reach upwards of 40 dyne/cm^2^ (see Supplementary Material), which per the above discussion is within a physiologically relevant range. This brings to light a potential limitation of inferring vessel WSS values based solely on visual observation of flowing RBCs, particularly for smaller capillaries.

While single per-vessel TAWSS values can provide a first approximation of the EC microenvironment during angiogenesis, the wide range of reported in vitro values that can elicit EC responses suggest more granularity is needed to pinpoint specific angiogenic behavior observed in vivo in response to specific hemodynamic forces. This is supported by other in vitro works that have shown strong connections between EC responses and TAWSS spatial gradients^61–64^ (ie, as opposed to single TAWSS values). However, given the difficulties of making such detailed measurements during angiogenesis in vivo, a critical first step in connecting in vivo EC responses to spatial gradients is to directly identify 3D TAWSS spatial patterns that can exist during angiogenesis, and hence a major focus of this work. The high-resolution simulations here have enabled quantification of these spatial variations at a highly localized level of detail. In terms of magnitude of variations, we have shown that the per-vessel TAWSS standard deviation can reach upwards of 55 dyne/cm^2^. Such variations are significant, given the aforementioned works, which have shown single TAWSS values themselves in this range to be physiologically relevant, let alone the spatial variations.

In addition to the magnitude of TAWSS spatial variations, we also quantified detailed maps of the TAWSSG directional components. The rich patterns further reveal and quantify the extent to which 3D spatial variations likely occur during angiogenesis in vivo. The existence of 3D spatial gradients over undulating surfaces of EC sheets was demonstrated in vitro by Barbee,^7^ who showed gradient hot spots in the flow direction can reach up to 7 dyne/cm^2^/µm. How ECs respond to such gradients has been reported by in vitro works,^61,62^ which showed how EC migration patterns and directions are connected to flow orientation. In vivo work by Franco et al.^65^ has also shown EC migration directional behavior to be influenced by flow direction. Such directional response of ECs emphasizes the importance of elucidating the directional components of 3D TAWSSGs, which can occur in real angiogenic vessel morphologies. Here we have shown that the TAWSSGs averaged over each vessel can reach 15 dyne/cm^2^/µm (axial) and 13 dyne/cm^2^/µm (circumferential), with a significant heterogeneity observed across all vessels. Further considering the 3D TAWSSG spatial patterns we observe, as well as maximum values that can reach 300 dyne/cm^2^/µm at highly localized surfaces within vessels (see Supplementary Material), a new picture is painted of the directional forces (and their magnitudes) likely experienced by ECs in vivo during angiogenesis.

Experiments showing connections between EC directional migration patterns and shear stress gradients have focused on flow direction gradients (ie, our axial direction). Yet, by modeling 3D vessels and RBC-influenced fluid dynamics our findings quantify significant circumferential gradients that can exist that are perpendicular to the flow direction. Earlier in vivo works in non-angiogenic microvasculatures, such as those by Kim and Sarelius,^90^ and Noren et al.^94^ showed how shear stresses can be different on opposite sides of vessels, which suggests the existence of circumferential TAWSS variations. This was especially noted in the latter of these works within bifurcations. We have shown and quantified here the existence of such spatial patterns in 3D, and not only within bifurcations but also along capillaries due to local surface undulations arising from vessel cross-sectional variations and centerline curvature changes. Importantly, we have shown that RBCs significantly enhance gradients in the angiogenic networks yielding values roughly 3–5 times greater than without considering RBCs, and that the circumferential component is increased by 5–10 times compared to the axial component. These findings emphasize the role played by 3D geometric complexity and individual RBCs in driving the complex force profiles experienced by ECs during angiogenesis in vivo.

Microvascular loop structures are also shown to experience regions of highly localized TAWSS spatial variations. We observe loops to typically have at least one vessel with negligible TAWSS values, naturally leading to sharp gradients at locations where such vessels connect to other vessels. Furthermore, we have shown how some loop structures can have vessels with multi-directional flow due to RBCs. Combined with patterns due to local 3D surface complexities, our findings generally reveal loop structures to be regions with significant TAWSS spatial variations. Recently, Ghaffari et al.^38^ reconstructed isolated microvascular loops in 2D based on in vivo images from the capillary plexus of avian embryos, and predicted TAWSS values ranging from negligible to 1.4 dyne/cm^2^ using a single-phase blood flow model. Notably, they observed the locations of TAWSS minima, excluding regions where flow streams merge, to generally correlate with locations of new sprout growth. While TAWSS variations in loop structures predicted in the current work are nearly two orders of magnitude larger, and despite differences in the nature of the angiogenic processes, these previous findings demonstrate the ability to connect TAWSS patterns to specific and highly localized angiogenic events in vivo. Thus, the new 3D behavior revealed here encourages developing new mechanistic understanding of EC movement during angiogenesis in vivo based on high-resolution 3D surfaces and RBC-resolved fluid dynamics.

Recent findings connecting sprout formation to regions of low TAWSS speak to the importance of identifying the existence of localized shear stress extremum in angiogenic microvascular networks along with influencing factors. We have shown here that 3D microvascular surface features and the presence of RBCs enhance both TAWSS hot spots and cold spots. Enhancement of cold spots by RBCs resulting in focused regions of lowered TAWSS (Figure 8) is a mechanism supporting our observation of RBC-enhanced TAWSS minima per vessel (Figure 6A-ii). TAWSS hot spots due to changing vessel curvature (Figure 8B) result in distinct patterns alternating between vessel sides, with lower TAWSS values occurring on the apices of tortuous vessels. Interestingly the latter is in agreement with recent in vivo work that connected in vivo vessel tortuosity to increased sprouting on the apical sides.^71^ Hot spot enhancement is also observed here due to the wrapping motion of RBCs flowing around pillar-like obstructions (Figure 8A), a geometry that bears resemblance to that reported by Arpino et al. in an in vivo study identifying such structures as integral to intussusceptive angiogenesis. The images of our angiogenic microvascular networks come from single points in time, and thus it is unclear whether the vessels in this region formed through intussusception. Nevertheless, our findings suggest asymmetrical shear stress patterns significantly enhanced by RBCs may influence behavior in angiogenic geometries of this nature.

Lastly, we have augmented the concept of WSS variations and heterogeneity by also analyzing variations in time. Examples of instantaneous WSS patterns corresponding to local RBC configurations and near-wall behavior suggest a causal role of RBCs on hot spots. This results in spatial patterns that move and evolve with the RBCs, and fluctuations in WSS statistics occur on timescales similar to that of individual RBC passage. To provide context for this, the overall physical time simulated is on the order of 1 s, during which time many RBCs (on the order of 1000) may pass by a particular wall location. This justifies our assumption of rigid vessel walls, considering the timescales of vessel adaptation are orders of magnitude longer. The same argument, however, suggests that the influence of the temporal variations reported here on angiogenic changes may be questionable. Nevertheless, we include the time-dependent behavior to provide a thorough description of the model predictions and disseminate findings.

Overall, our findings reveal a new high-resolution 3D picture of the microenvironment likely underlying angiogenic processes in vivo. This advancement provokes new questions to understand roles of 3D shear stress patterning on EC behavior along vessels. Such provocation motivates future studies correlating the presence of 3D shear stress spatial and temporal patterns with patterns of EC phenomena, such as local hot spots and EC permeability or junctional disruptions, or spatio-temporal variations and signaling coordination.

### Limitations and Additional Considerations

Appreciation of the presented findings requires discussions on the modeling limitations. The many big-picture-type challenges associated with getting the model “right” provoke consideration of the model’s purpose. The challenges and assumptions with this type of modeling include aspects such as selection of boundary conditions, initial placement of RBCs and injection at inlets to maintain hematocrit levels, resolving 3D geometrical features, or necessary compromises to manage long run times. A model is only as good as what it is used for, and for the current work, our goal is to identify potential shear stress patterns in remodeled network regions post angiogenesis taking into account features such as 3D fluid dynamics, RBC deformation and interactions, vessel connectivity, and vessel specific curvatures. The discussions below emphasize many of the challenges in achieving this, how we have overcome them and resulting limitations, and provide context for interpretation of presented findings.

Boundary conditions for the simulations are prescribed flow rates at the inlet and outlet vessels. While we do not know what the actual boundary conditions should be, the flow values were chosen to yield shear rates spanning a physiological range,^81^ as discussed in Materials and Methods. Similarly, hematocrit values at inlets were prescribed following physiological values reported for the microcirculation.^81,82^ Given the known heterogeneity of hemodynamic quantities in the microcirculation, parameter selections that are also physiological could ostensibly result in increased/decreased shear stress magnitudes and patterns beyond what is reported here. Yet, values were chosen to span an “average” physiological range based on literature values and thus results provide a representation in this context of what may occur during angiogenic remodeling.

The choice of flow versus pressure boundary conditions was specifically made to facilitate shear stress comparisons between RBC-resolved simulations and plasma-only simulations. Fixed pressure boundary conditions would result in similar per-vessel WSS values between the RBC and plasma-only simulations due to resulting similar per-vessel pressure drops based on the fluid mechanics, as observed in prior work.^8^ On the other hand, flow boundary conditions have enabled isolating contributions of RBCs to shear stress behavior. Fixed pressure boundary conditions could be used toward understanding flow distribution patterns and connections to hydrodynamic resistance, although we limit our focus here to the WSS alone. We also note that, even though we have flow boundary conditions, when we change them (either by the 0.5x or 2x factors) the per-vessel TAWSS throughout networks does not just linearly scale in proportion to the boundary flows. This is due to the presence of the RBCs. Figure 7B shows how, if you have just plasma and you double or halve the prescribed flows at the boundaries, the shear stresses in all of the vessels scale in proportion. In contrast, if you have RBCs this is not necessarily the case, especially in smaller-diameter vessels.

The initial shapes of RBCs injected at inlets are deformed and highly asymmetric and derive from separate straight tube simulations resulting in random placement over inlet cross-sections. After initial RBC injection, interactions with other RBCs as well as the complex vessel geometries quickly dominate RBC motion and dynamics. Different inlet positions of each injected RBC would likely affect the specific time-dependent WSS patterns in vessels, however, it is not expected that such differences would alter any of the main findings presented.

The physical dimensions of the modeled RBCs are consistent with human RBCs,^74^ while the angiogenic microvascular networks considered are from the rat mesentery. The ultimate goal of this work is to understand and predict representative shear stresses during angiogenesis, and what we really care about is the human scenario. To this end, we modeled human RBCs, and the rat mesentery is justified because it displays characteristics of angiogenic networks regardless of species. In terms of particle-scale effects on shear stresses, we note that human and rat RBC dimensions differ in their major radius by approximately 0.4 µm and that human RBCs have ∼30% greater surface area and 50% greater volume.^74,75^ This potentially would result in a greater impact of human RBCs on shear stress than rat RBCs. Further, based on these size differences, the number of rat RBCs required for a specific hematocrit level would be increased over human, but not by an order of magnitude. While different specific time-dependent WSS patterns would likely occur, the qualitative details of the representative patterns shown here would not be fundamentally different.

The time-dependent WSS behavior reported here occurs entirely because of the deformable RBCs, as the boundary conditions are fixed. This results in relatively high-frequency WSS oscillations (∼0.01 s) compared to larger-scale oscillations (∼1–100 s) due to various changes in vessel diameters attributed to local smooth muscle cell reactivity along arterioles, pre-capillary sphincters,^95,96^ or even pericyte regulation along capillaries.^97^ Additionally, work by Kiani et al.^98^ both computationally and experimentally showed how RBCs themselves can produce oscillations in vascular networks with timescales in the range of ∼5–25 s. This warrants future consideration of RBC-resolved simulations at these longer timescales to investigate resulting oscillations and effects on WSS. The three different boundary flow strengths considered in the current work provide some measure of varying WSS responses to flow conditions, but the dynamics of transitioning between flow states is not known. While the current work isolates temporal fluctuations due to RBCs, interrelated effects of fluctuations due to other sources, both at vessel and sub-vessel levels, remain to be investigated. We also note that for each of the three flow conditions considered for each network, the geometries remain fixed. For such scenarios, the predicted per-vessel values across these conditions are altered such that both the average shear stress values and the heterogeneity increase with flow strength (Figure 7A). In addition, the distribution of shear stress values among the vessels is altered in a non-linear fashion due to the presence of RBCs (Figure 7B). For other scenarios in which the simulated network diameters may change, this would likely alter the heterogeneity as well, although this remains to be investigated as well.

Due to the computational effort involved, simulations span a physical time on the order of 1 s. Effects of oscillations at the longer timescales noted above are difficult to estimate. On one hand, due to the flow regime it might be justified to assume the behavior can be captured by a superposition of separate simulation results at different, steady boundary conditions. Yet on the other hand, due to the presence of RBCs, and considering changes in microvascular tone/structure over time, there are non-linear effects present that could influence the behavior during microvascular transitions. An important point to note though is that, in a broad sense, what this work is highlighting is localized 3D heterogeneity. If one were to consider a 1 s window 10 min later, the specific results might be different, but this heterogeneity will still exist. The real extent of the spatiotemporal heterogeneity though, is unknown. So, while we do not know exactly if or how such oscillations will affect behavior, future investigations are warranted.

Vessel tortuosity is a hallmark angiogenic microvascular characteristic, and here we have shown how shear stress hot spots form in tortuous vessels near regions of high surface curvature (Figure 8B), similar to other recent work.^8^ Specific characteristics of tortuous vessels are expected to vary based on location in the body and function. The findings here provide evidence that the angiogenic tortuous vessel characteristic results in distinct 3D shear stress patterns and warrants future investigation to identify tissue and organ-specific behavior. For example, in skeletal muscle with increased vessel tortuosity in conjunction with rapidly changing RBC fluxes, there may be unique enhancements to shear patterns to achieve a functional end, although future investigations are needed.

For the current study, the selection of angiogenesis stimulation was arbitrary; given the study’s focus on modeling RBC flow through angiogenic networks, rationale for the use of compound 48/80 stimulated tissues is supported because the networks display angiogenic remodeling characteristics. While the specific mechanism remains unknown, the stimulation has been associated with mast cell release of histamine and other highly active substances.^84,85^ It is unknown whether WSS is a triggering mechanism. However, based on these previous studies, we speculate that angiogenesis is initially triggered by changes in the local chemical environment. An intended main takeaway of the current study is the identification of potential RBC flow dynamics, including local WSS spatiotemporal patterns and the use of the actively remodeling network regions enables this. The representative regions used for the current study were selected because they displayed high vessel density and patterns reflective of active network remodeling. Regions of interest did not include capillary sprouts. Hence, the vessel patterns in the angiogenic regions can be assumed to be post the initial capillary sprouting stage. Future studies will be needed to evaluate RBC flow effects due to sprout presence, sprout dynamics, and sprout connection with other vessels. The lack of sprouts in the regions for the current studies emphasizes that our results provide insights into the effect of remodeling on local RBC flow dynamics versus the implication of RBC flows on sprouting. Further, our results motivate similar future studies to determine whether similar RBC flow dynamics are associated with angiogenic patterns due to other alternative stimuli and across different tissue beds.

This work focuses on the shear stresses experienced due to hemodynamics and does not include other factors known to contribute to angiogenesis, such as tissue growth factors, interstitial transport, or transendothelial transport. Following the discussion above related to time-dependent behavior, the timescales resolved here are of a different order than those associated with these processes, and thus impacts on our findings are expected to be small. In terms of 3D vessel surface features, the model also does not account for the endothelial surface layer (ESL) or individual EC surface structures. 3D networks were constructed assuming locally circular lumen shapes and do not resolve asymmetrical cross-section shapes or surface irregularities. While such considerations will likely influence the exact 3D shear stress patterns, here we have focused on revealing types of patterns and behavior that can occur when 3D angiogenic vessel structures and RBCs are resolved.

The findings suggest that including individual EC geometries or other angiogenic surface irregularities may lead to unique 3D shear stress patterns. For example, vessels with changing cross-sectional shapes and out-of-plane asymmetries may result in an increased frequency of local hot and cold spots, especially where more severe local surface variations occur. This is supported by the patterns we have shown in Figure 4B near the red and blue arrows, which are similar in spirit to this. Additionally, inclusion of individual EC shapes may result in sharp WSS gradients at locations where ECs connect to one another, due to locally high surface curvatures near EC junctions. In the current work, high curvature of local surfaces such as Figure 8B (feeding vessel), resulted distinct hot spots and large local surface gradients. Going forward, the specific details and idiosyncrasies of such patterns are difficult to predict without explicitly modeling them. We have shown here that revealing such details requires high-resolution RBC-resolved simulations, yet in the future, the output from such simulations could in principle be used to build improved reduced-order models and circumvent the need for large-scale simulations. Altogether, the identification of the representative patterns presented here is an important first step needed to inspire new research directions for vascular biologists and modelers alike to tackle ideas such as ESL/EC shape influences.

## Supplementary Material

zqad046_Supplemental_File

## Data Availability

All data needed to evaluate the conclusions in the paper are present in the paper and/or the Supplementary Materials. Data and computer code files can also be accessed via the Supplementary Materials, which include links to the repositories where the information is stored.
