# Supplementary material for: Angiogenic Microvascular Wall Shear Stress Patterns Revealed Through Three-dimensional Red Blood Cell Resolved Modeling
Source: Function (Oxf). 2023 Aug 29;4(6):zqad046. doi: 10.1093/function/zqad046 (PMC10519277; doi:10.1093/function/zqad046)
Supplement: zqad046_Supplemental_File [file zqad046_supplemental_file.docx]

**Supplementary Materials**

**Angiogenic Microvascular Wall Shear Stress Patterns Revealed Through Three-Dimensional Red Blood Cell Resolved Modeling**

Mir Md Nasim Hossain^1^, Nien-Wen Hu^2^, Maram Abdelhamid^1^, Simerpreet Singh^1^, Walter L. Murfee^2^, and Peter Balogh^1^*

1. Mechanical and Industrial Engineering, New Jersey Institute of Technology, Newark, New Jersey, U.S.A.

2. J. Crayton Pruitt Family Department of Biomedical Engineering, University of Florida, Gainesville, Florida, U.S.A.

*peter.balogh@njit.edu

**Movies of RBCs Flowing Through Angiogenic Microvascular Networks**


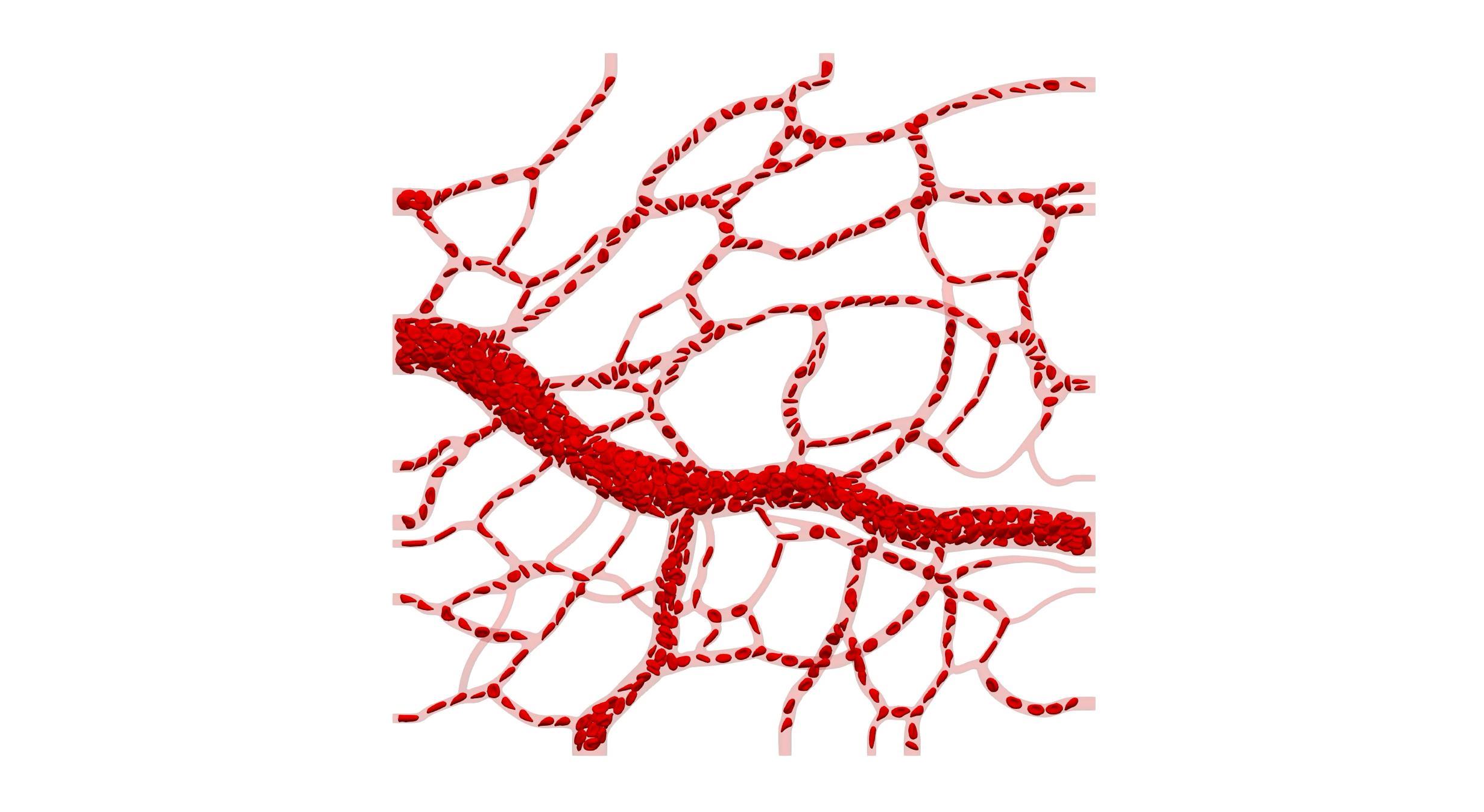


**Movie S1:** Flow of RBCs inside the first angiogenic network, movie can be accessed [here](https://drive.google.com/drive/folders/1Wb4SpYOM9muX5hjOeOaWaU55lbiPux5C?usp=share_link). NOTE: Playback is not real-time. Overall duration corresponds to roughly one second of physical time.


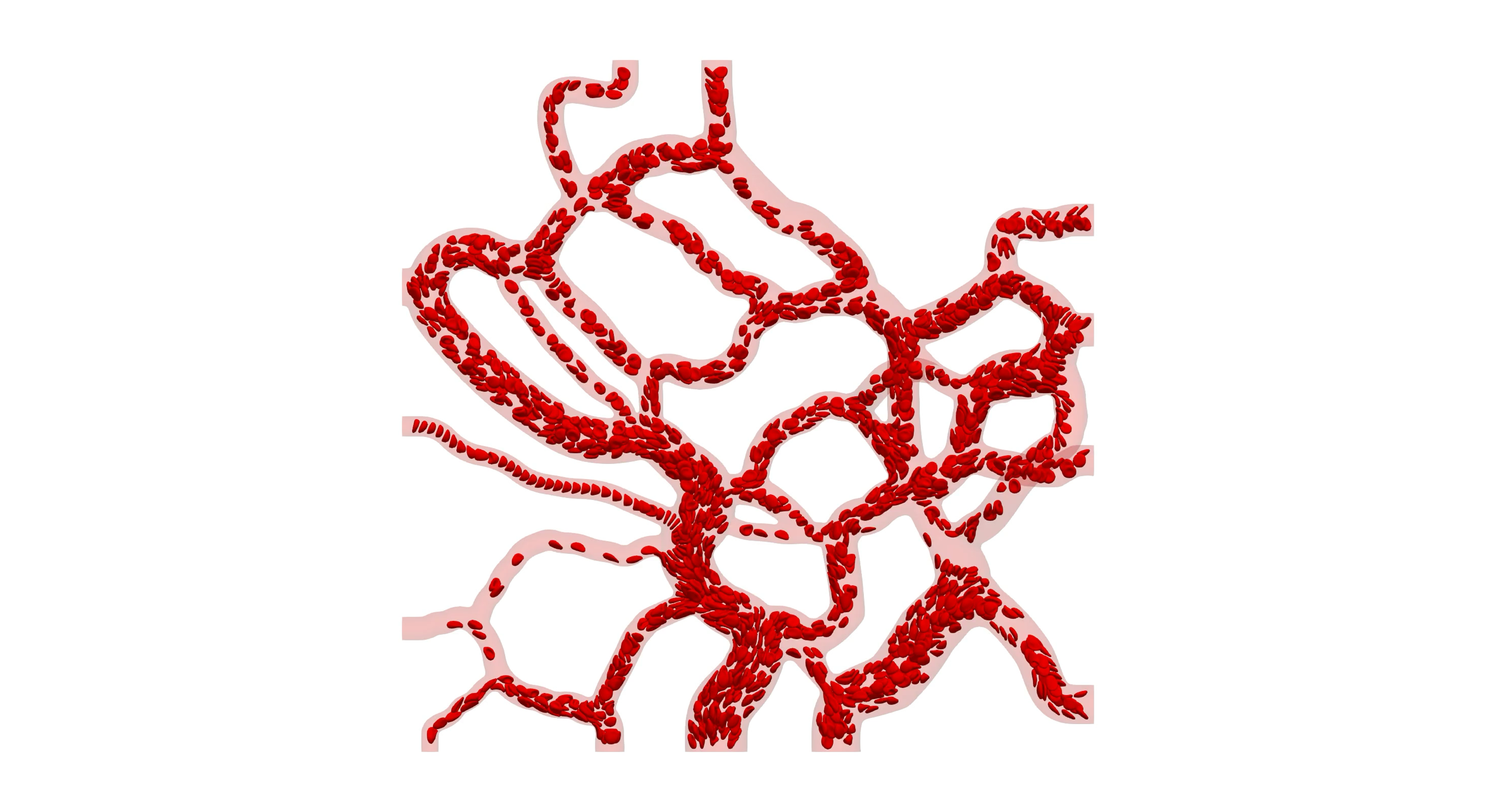


**Movie S2:** Flow of RBCs inside the second angiogenic network, movie can be accessed [here](https://drive.google.com/drive/folders/1Wb4SpYOM9muX5hjOeOaWaU55lbiPux5C?usp=share_link). NOTE: Playback is not real-time. Overall duration corresponds to roughly 0.75 seconds of physical time. On the left side border and near the top, there is a discontinuity in the geometry associated with this very short segment (L/D << 1) being a former inlet. This region is not included in any analysis.


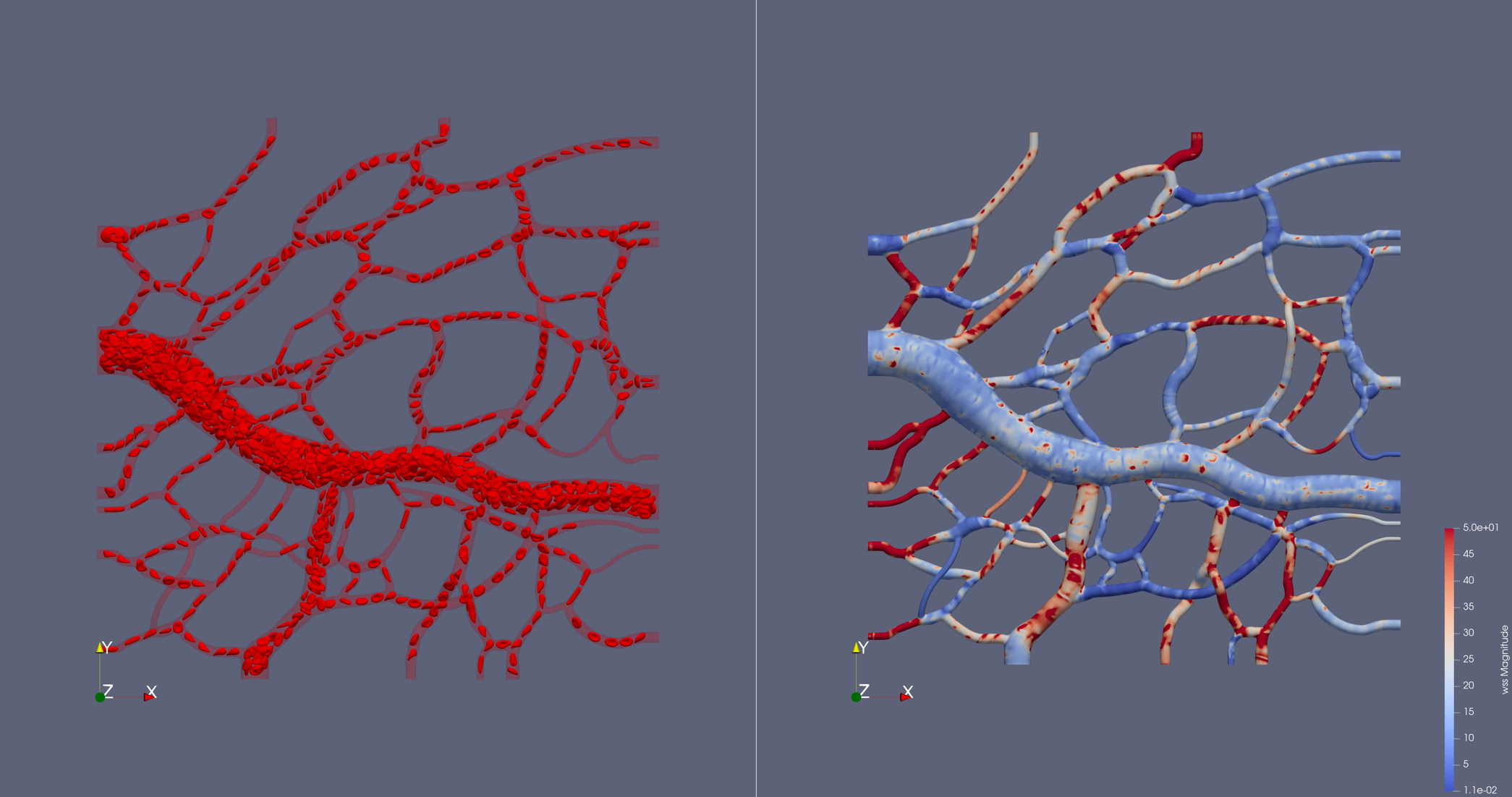


**Movie S3:** Instantaneous change of WSS due to RBCs flow, movie can be accessed [here](https://drive.google.com/drive/folders/1Wb4SpYOM9muX5hjOeOaWaU55lbiPux5C?usp=share_link). NOTE: Playback is not real-time. Rate is the same as with Movie S1.

**Multidirectional flow of RBCs inside a loop vessel**

A unique behavior of loop is that on the cross stream of bifurcation some vessels show multidirectional flow. A movie is shown in Movie S4 for such a loop where RBC flow in both directions depending on time. Before flowing in any direction RBCs linger a small amount of time and then flow in both directions based on time and surrounding RBCs flow. There is significant effect of surrounding RBCs on the lingering RBC on the flow direction at any time instant. At any moment when a RBC is lingering on the multidirectional vessel, that changes the hydrodynamic resistance instantly on the surrounding region. So, the following RBCs flow on the vessel that gives less resistance to flow.


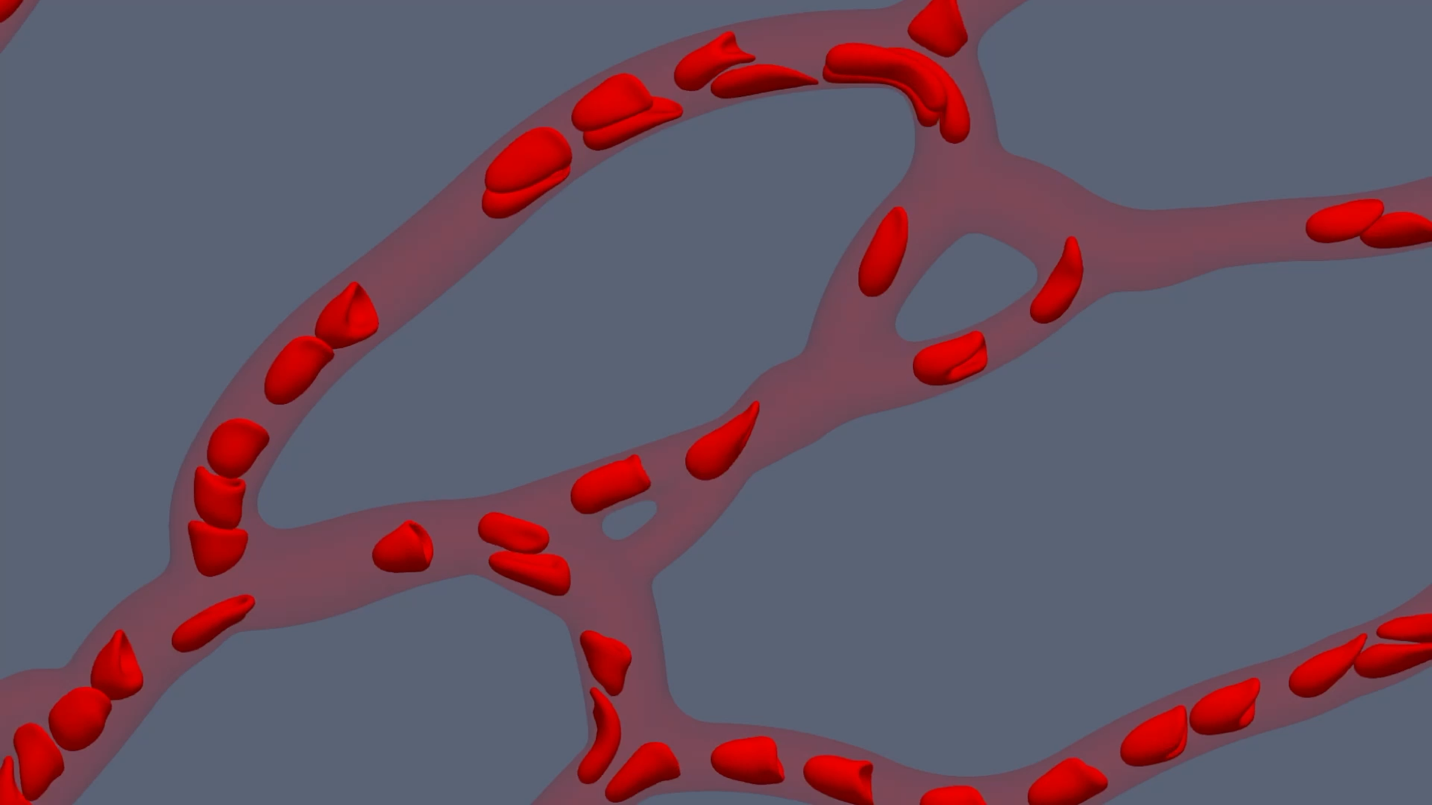


**Movie S4:** A multi directional flow vessel, movie can be accessed [here](https://drive.google.com/drive/folders/1Wb4SpYOM9muX5hjOeOaWaU55lbiPux5C?usp=share_link). NOTE: Playback is not real-time. Rate is the same as with Movie S1.

**Effects of shear rate on TAWSS and viscosity**

Figure S1(A) shows that TAWSS increases in direct proportion to shear rate. Shear rate is the effective shear rate for each vessel, calculated based on average velocity per vessel and vessel diameter. Figure S1(B) gives shear rate versus viscosity per vessel, where viscosity decreases with shear rate which shows the shear thinning behavior of blood.

Apparent viscosity values per vessel were calculated from the simulation data based on the TAWSS for each vessel. Namely, for a vessel $\mu_{app}=\mathrm{TAWSS}\cdot\frac{\pi D^{3}}{32Q}$, where $\mathrm{TAWSS}=\frac{\Delta P\cdot D}{4L}$ (from force balance) which connects to Poiseuille’s law. We note that these values are not used with any of the wall shear stress calculations that are the main focus of this work. With the 3D RBC-resolved simulations here, especially in the smaller diameter microvessels and the morphologies associated with the complex 3D regions where vessels connect (and in some cases very short vessels connecting to one another), computing an accurate measure of apparent viscosity in an individual vessel can be complicated, because such conditions represent a significant departure from homogenous fluid flow in a constant diameter tube. Nevertheless, we include this data here in an attempt to provide some connection between simulation predictions and common concept of blood viscosity (i.e. that associated with a Poiseuille-type flow in a straight tube).

**Figure S1:** Change of TAWSS and viscosity with Shear rate (A) Shear rate versus Time averaged WSS in logarithmic scales (B) Shear rate versus viscosity in logarithmic scale. In both cases each dot represents a single vessel.

**WSS variations within a low flow loop**


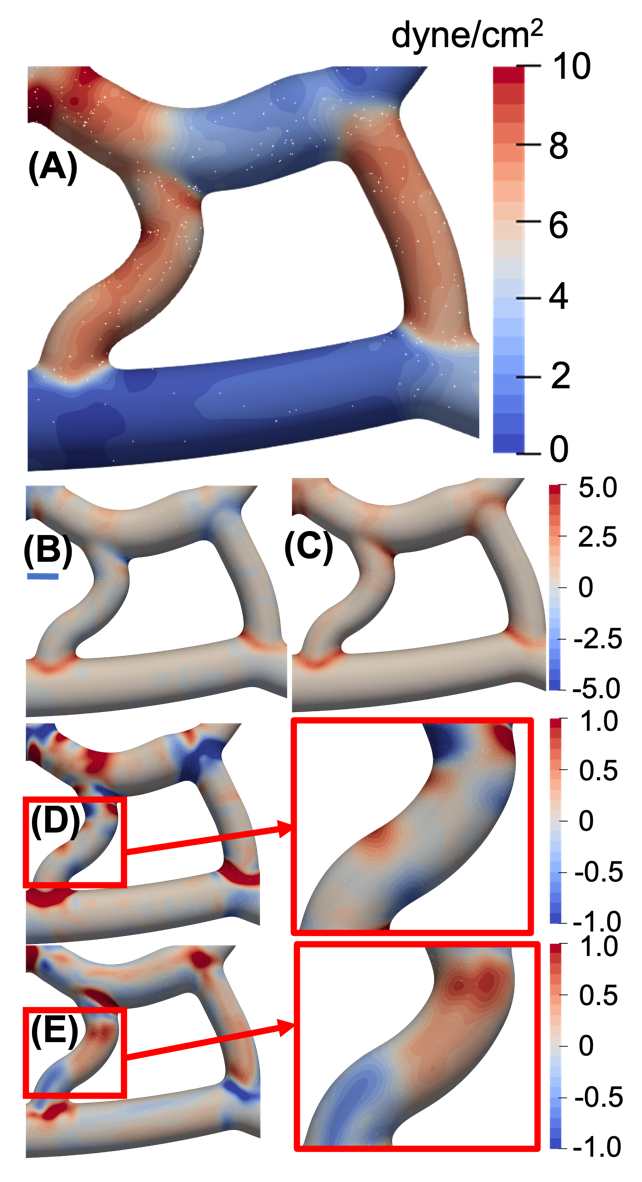
Low flow capillary networks also show significant WSS spatial variation which can be appreciated through adjustments to the visual scale. Figure S2 shows a representative low flow zone consisting of four capillary vessels. Maximum flow rate is 0.025988 mm3/hour with the velocity 0.2842317 mm/sec, which is significantly smaller than observed in the other vessels. Yet, rich WSS variations can still be observed as in the figure S2(A). The corresponding variations in WSSG are shown in the remaining sub-figures. From the magnified picture of a vessel presented in fig. S2(D) several hotspots and cool spots show significant spatial variation which is not noticed in large scale range. For each hotspot, a cool spot is generated at the opposite side of the vessel. These hotspots and cool spots are generated due to the curvature change of the vessel wall. Similarly for circumferential components of WSSG in fig. S2(E), significant spatial variation is noticed in smaller scale range.

**Figure S2:** TAWSS and TAWSSG in a low flow capillary loop. (A)TAWSS contour map for the same region in the range of 0 to 10 dyne/cm2 (B),(C) axial and circumferential component of WSSG respectively with cut off -5.0 to 5.0, Scale bar is 5 μm (D),(E) Axial and circumferential component of WSSG respectively of the same loop with cut off -1.0 to 1.0.

**Characteristic Microvascular Loop Behavior**

Angiogenic networks contain significant number of loops that makes it different from fully developed microvascular networks. Loops consist of 3 or more vessels which circulate and partition RBCs in multiple directions. We presented some loops found in the representative angiogenic networks along with TAWSS contour map in fig. S3. Almost all of them consist of at least one negligible RBCs flowing vessel. A qualitative analysis has shown that total 25 loops are present cumulatively in both angiogenic network among which 17 (68%) of them shows this behavior while some of them show multidirectional flow vessel. Negligible RBCs flow doesn’t mean that no flow occurs in that vessel, rather comparing other vessels flow of RBCs is less in the negligible flow vessel. Physiologically it is important because negligible flow vessels give RBCs more time to exchange nutrients and oxygen while other vessels pass the RBCs to subsequent vessels.

**
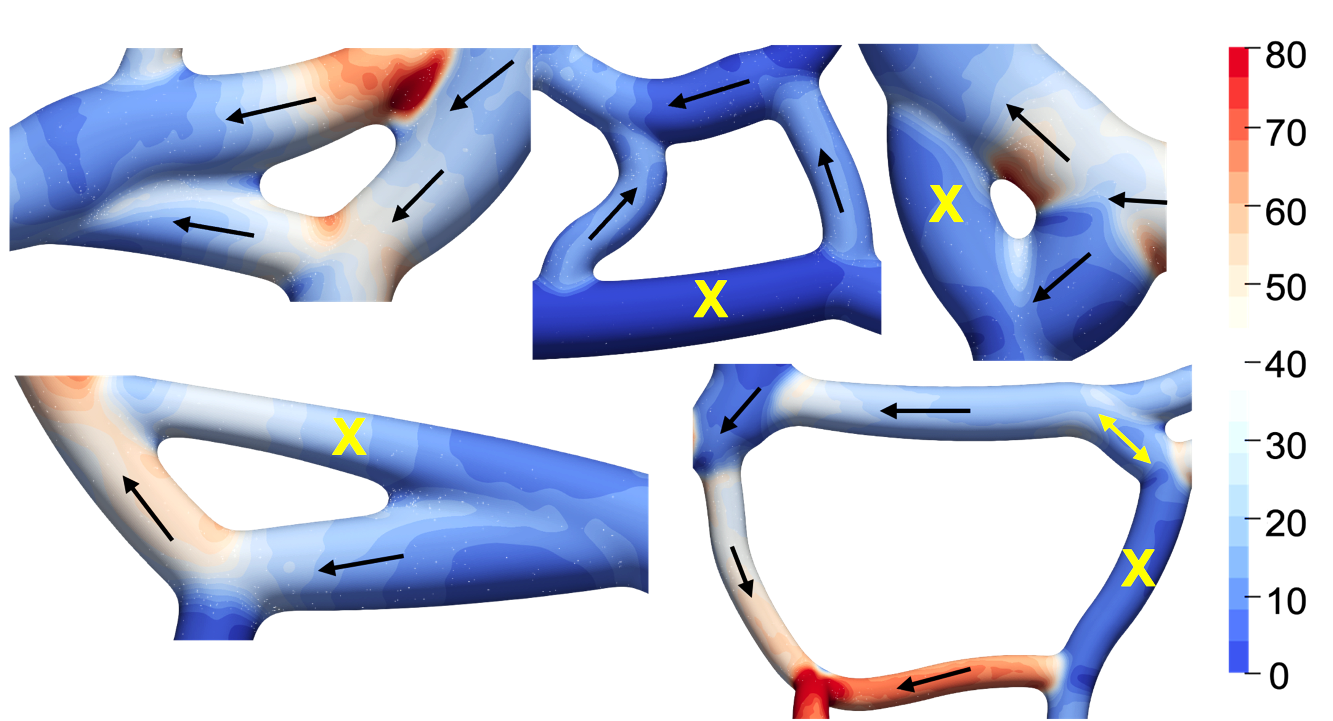
**

**Figure S3: Representative WSS contour of loops in angiogenic networks.** Black arrow gives flow direction, yellow arrows denote multidirectional RBC flow while yellow X denote a negligible RBC flow vessel.

**Effect of RBCs around pillar like obstruction**

Area around a pillar like obstruction is magnified and presented to compare pure plasma flow with RBCs flow in fig. S4. Red arrow shows a hot spot region which is enhanced five times in terms of magnitude because of RBCs in the fig. S4(B). RBCs come directly, stuck, and linger at that point momentarily which greatly enhances the WSS hot spot. See Movie S5 (accessed here) which shows an example RBC wrapping around the pillar. For plasma only simulation, due to absence of RBCs velocity gradient is not changed significantly to give a high WSS. Besides arrow directed region, other regions also show WSS changes of WSS in magnitude and area in the representative image. RBCs entirely flow through one vessel due to the geometric condition which increase TAWSS of that vessel. Overall spatial variation increases in the presented WSS contour due to RBC flow.


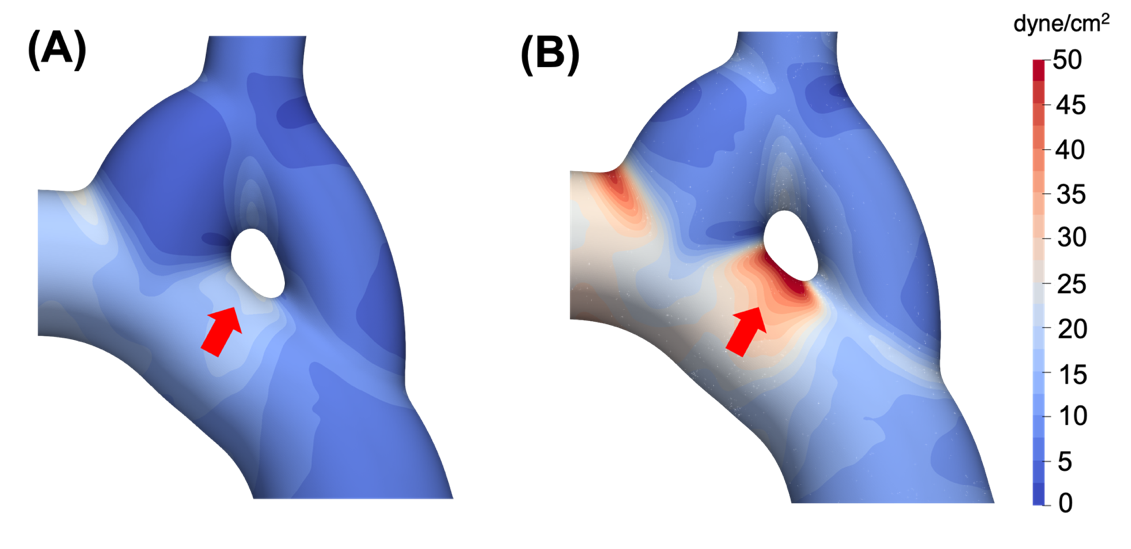


**Figure S4:** Effect of RBCs on TAWSS in a pillar like obstruction (A) Plasma only flow (B) Flow with RBCs

**High TAWSS in an RBC deprived vessel**

A specific region is magnified from the representative angiogenic network and both TAWSS contour map along with RBC snapshot is shown in fig. S5. Almost all the vessels contain RBC except one vessel that doesn’t have any RBC inside. Still TAWSS contour map from the fig. S5(B) shows high time averaged WSS (40 dyne/cm2) in that vessel which cannot be attribute toward the only RBC flow. Though similar value of TAWSS is observed in several other vessels presented in that region, it is obvious that RBC is not the only reason for high TAWSS.


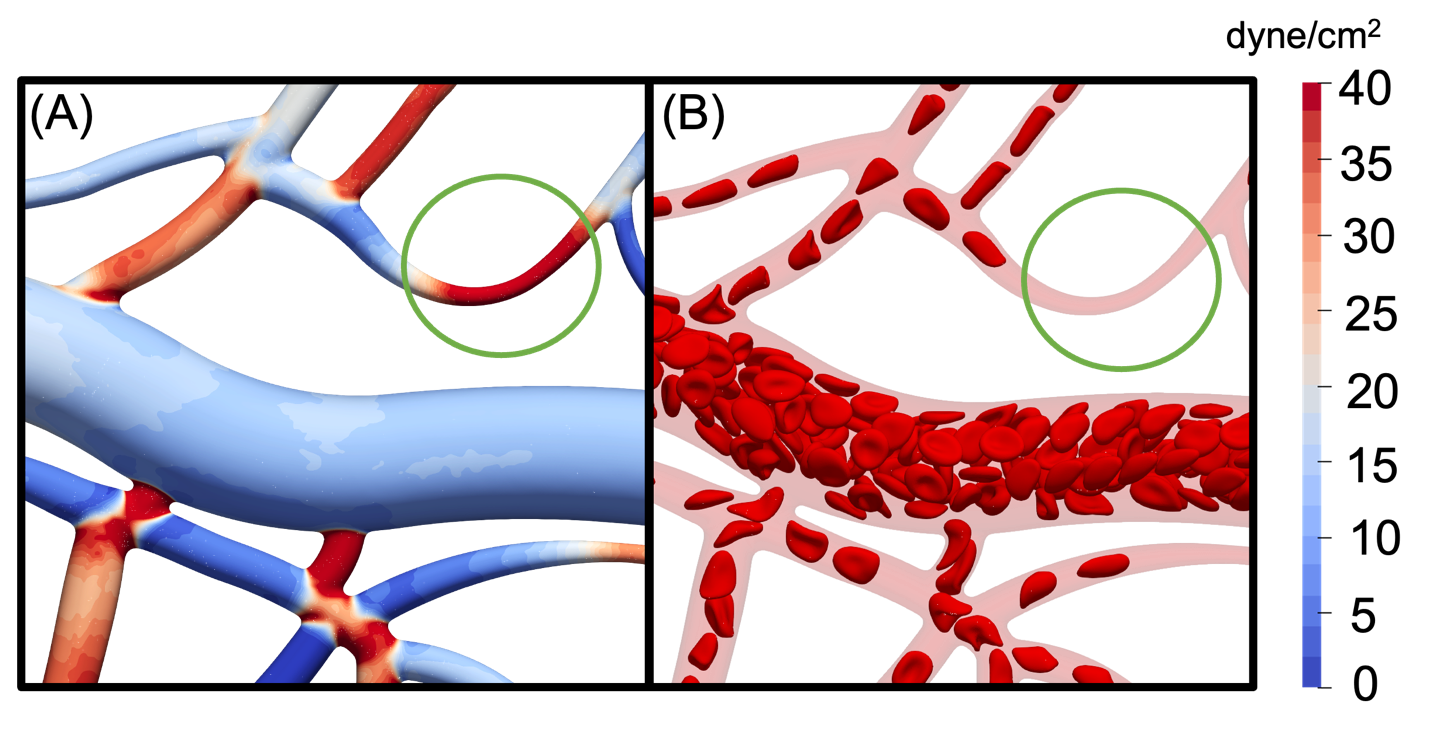


**Figure S5:** Presence of high TAWSS in RBC deprived vessel (A) TAWSS contour map in a specific region, (B) RBC snapshot of the representative area. For both cases green circle shows a vessel having no RBC on it.

**Maximum TAWSS per vessel**

Maximum value of TAWSSG is presented in fig. S6 for each vessel against vessel diameter. For most vessels, maximum is below 90 dyne/cm2 /µm while some of them has high value like 300 dyne/cm2 /µm. For low diameter vessel, high gradient value spread out more in the range of 0 to 300 dyne/cm2 /µm, but for high diameter vessel range is less which is from 0 to 30 dyne/cm2 /µm.


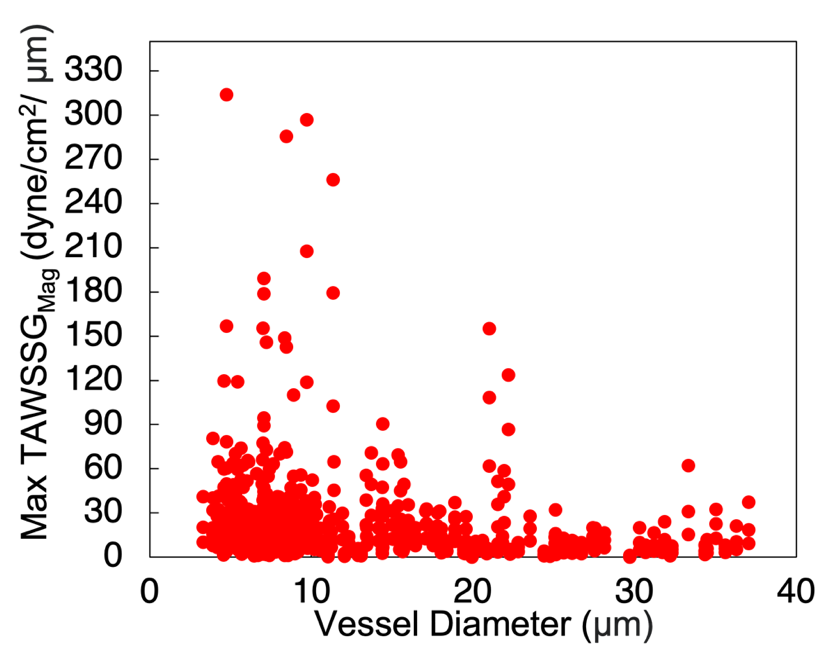


**Figure S6:** Maximum TAWSSG magnitude per vessel. Each dot represents the maximum value obtained at each vessel over the whole simulation time.

**TAWSS and TAWSSG Maps for All Simulations**


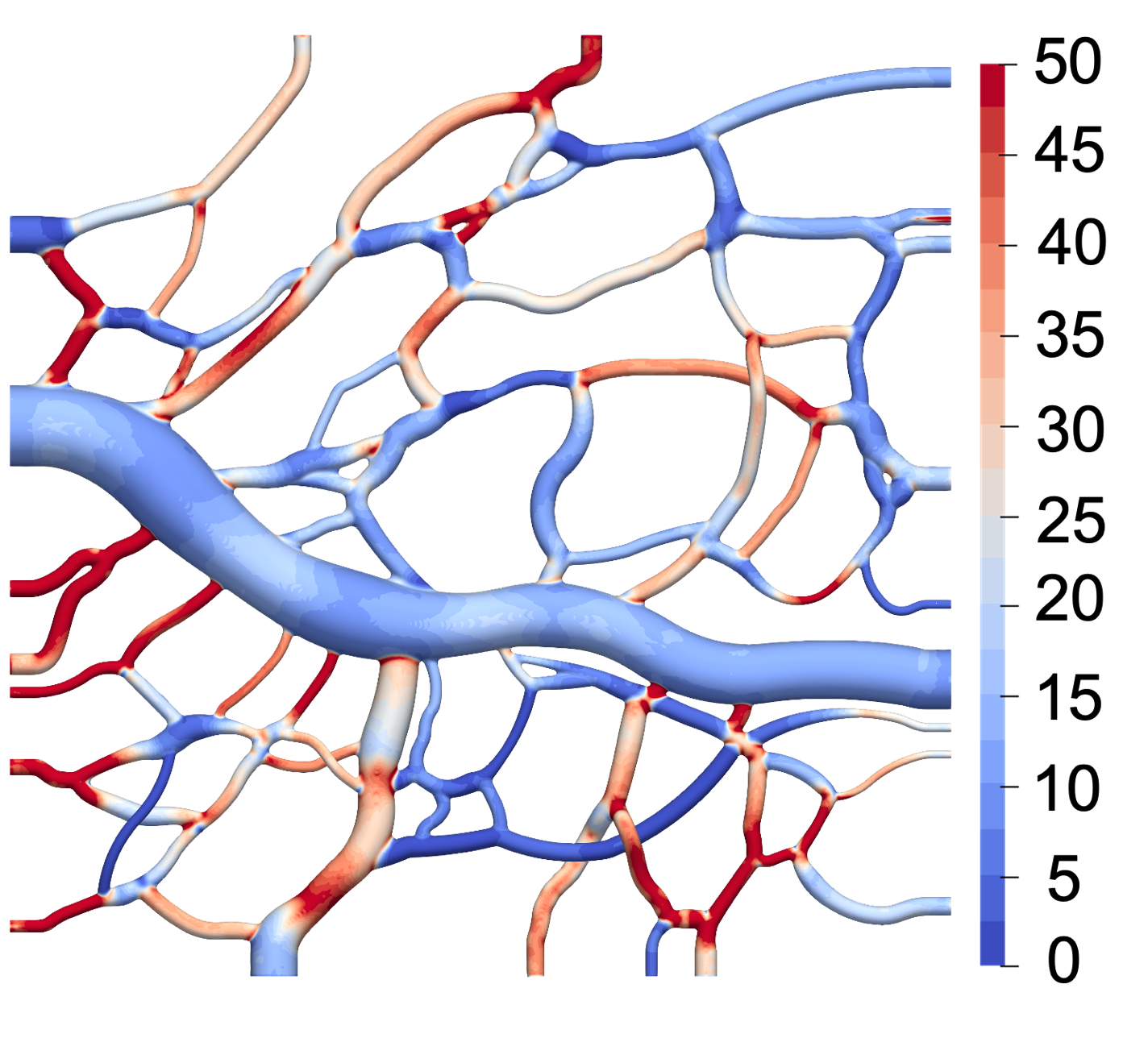


**Figure S7: TAWSS contour map of the first representative angiogenic network in base flow condition. Units are dyne/cm^2^.**


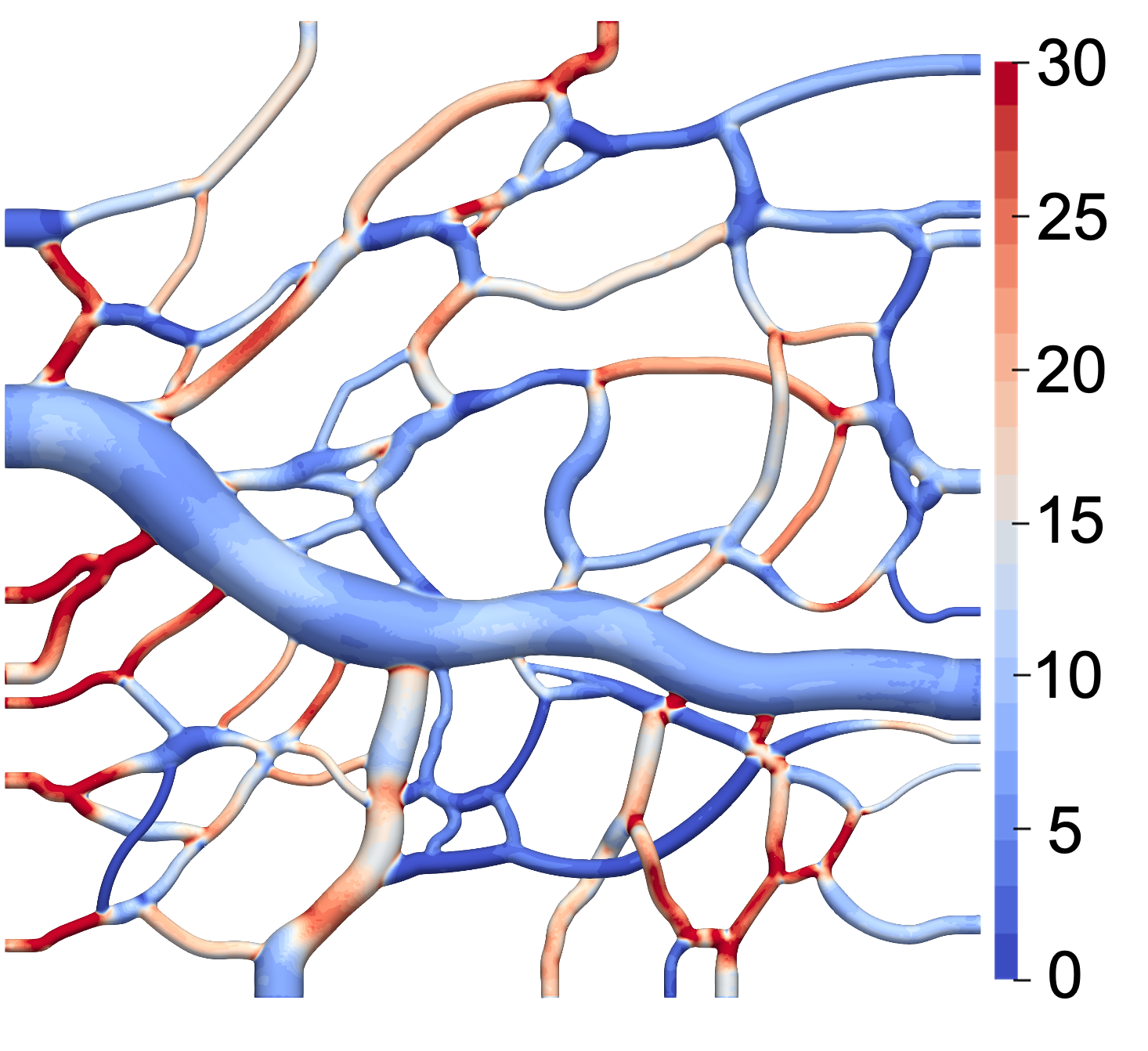
**Figure S8: TAWSS contour map of the first representative angiogenic network with 0.5 times of base flow condition. Units are dyne/cm^2^.**


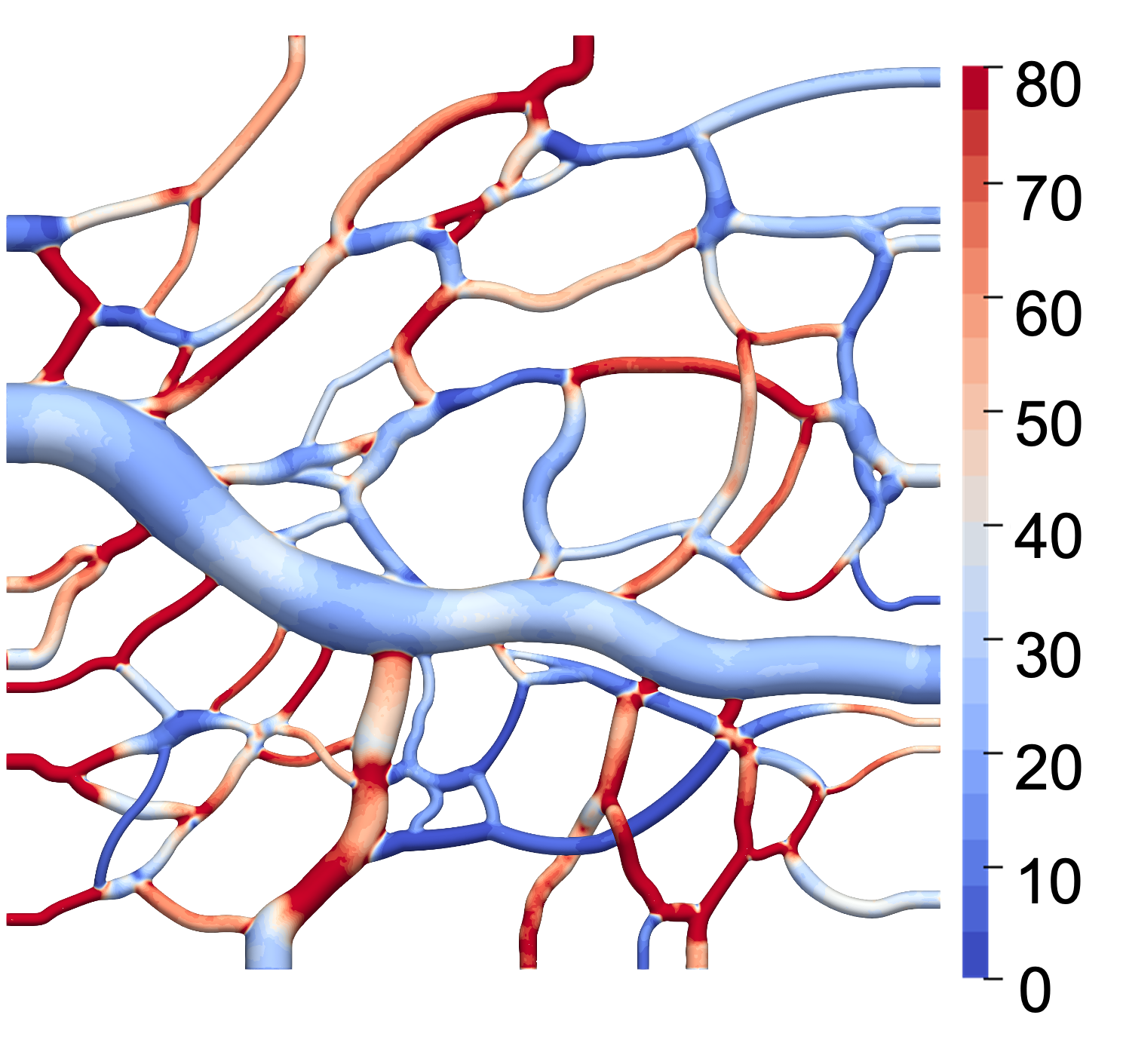


**Figure S9: TAWSS contour map of the first representative angiogenic network with 2.0 times of base flow condition. Units are dyne/cm^2^.**


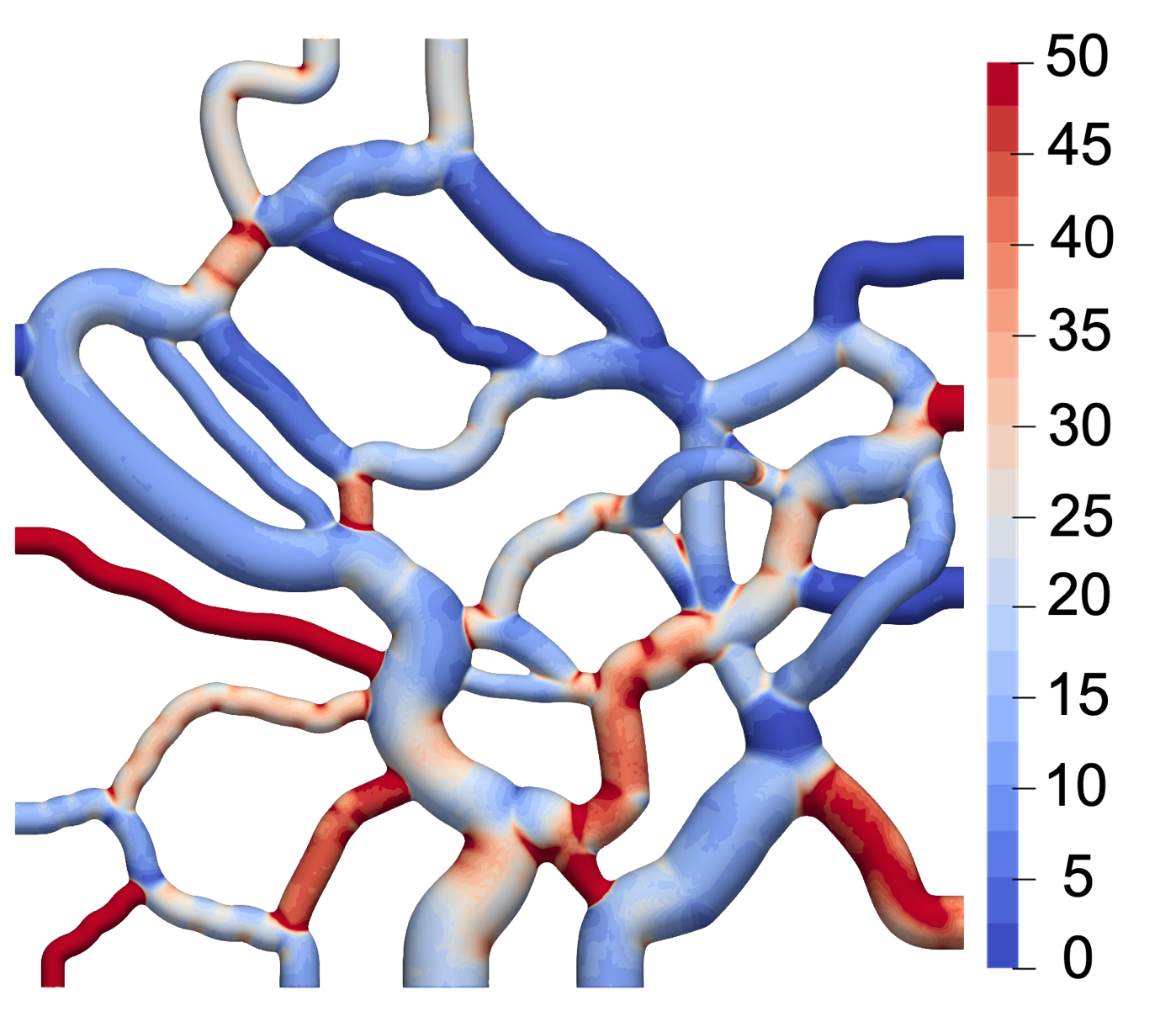


**Figure S10: TAWSS contour map of the second representative angiogenic network in base flow condition. Units are dyne/cm^2^.**


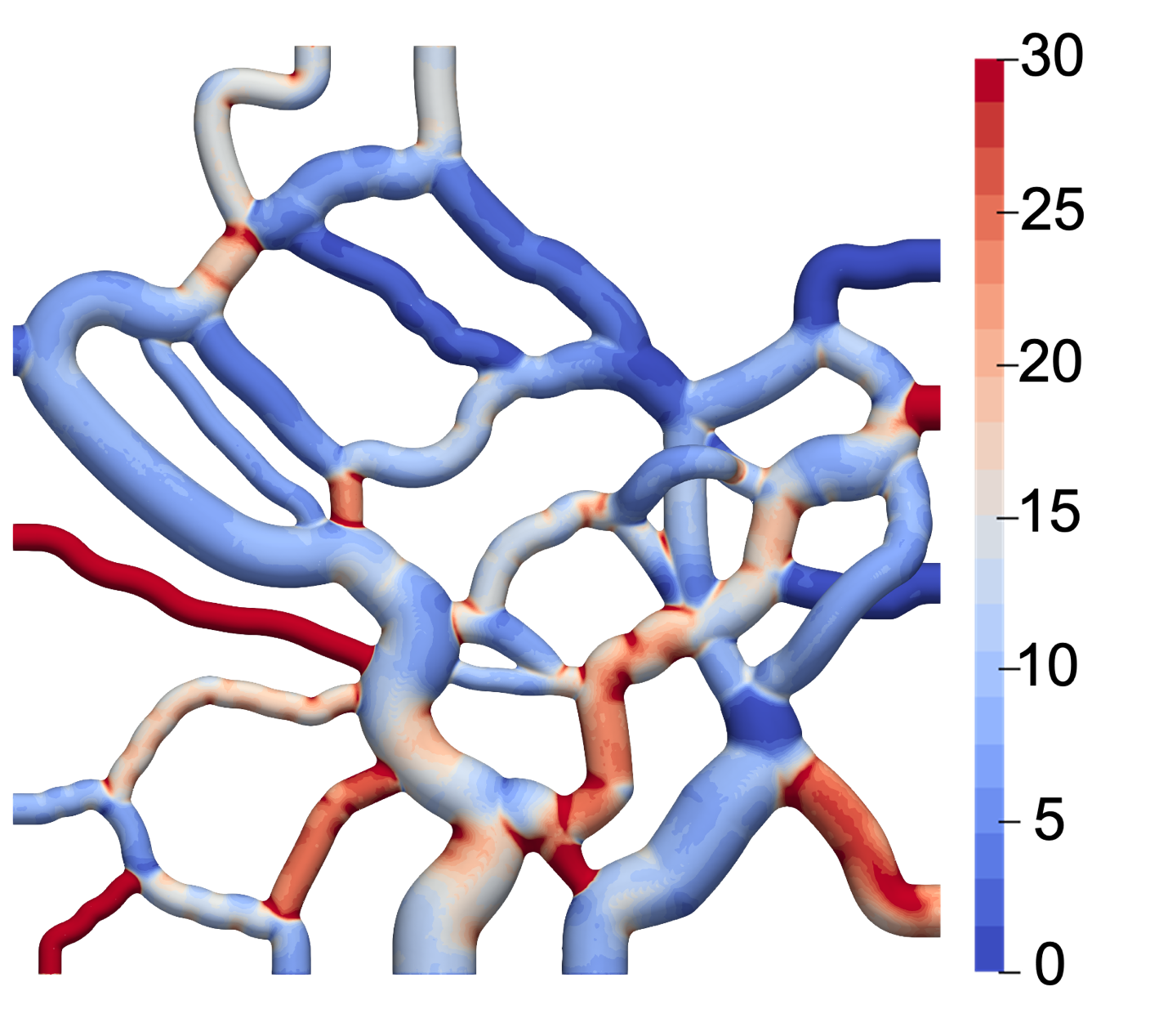


**Figure S11: TAWSS contour map of the second representative angiogenic network with 0.5 times of base flow condition. Units are dyne/cm^2^.**


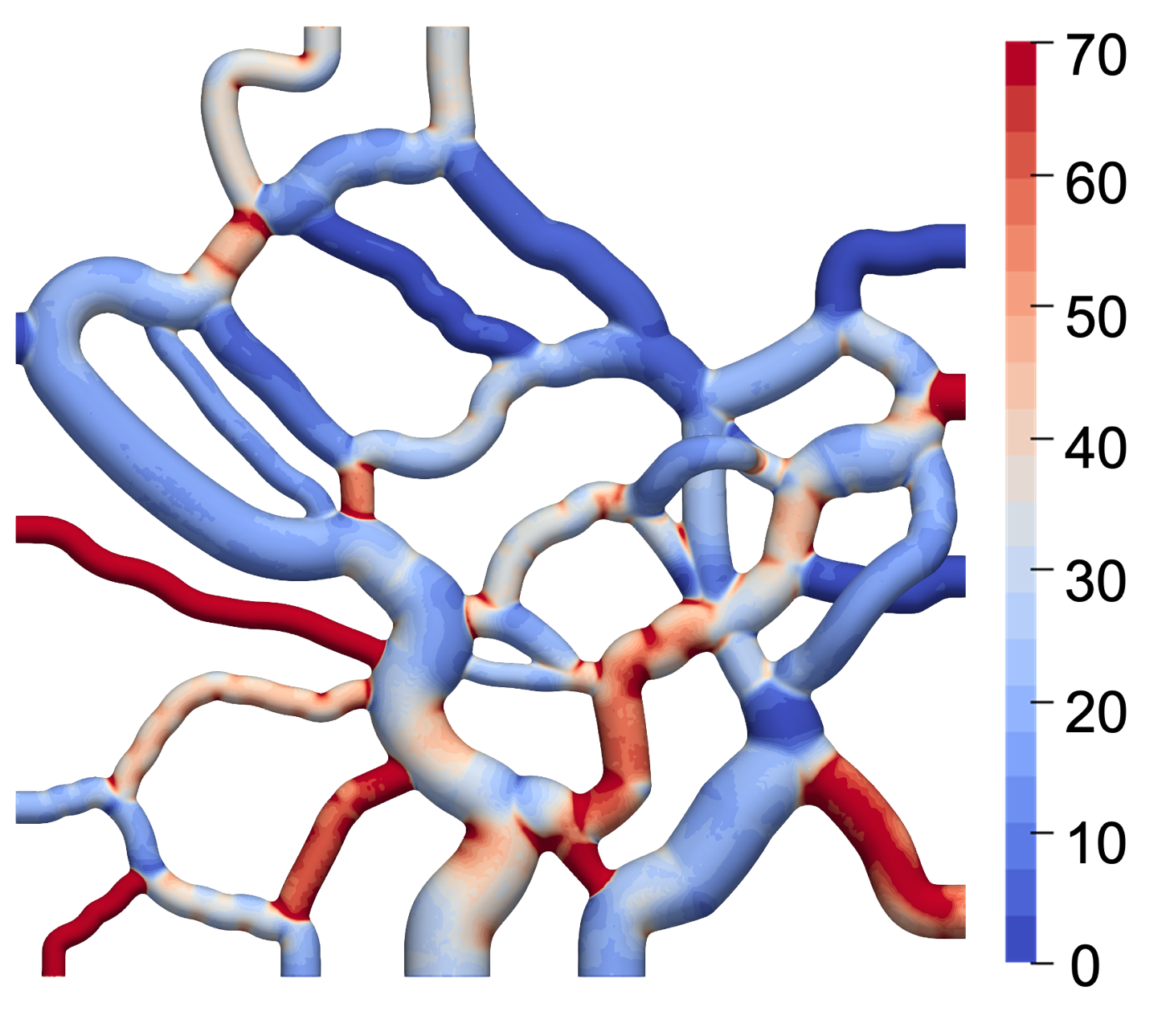


**Figure S12: TAWSS contour map of the second representative angiogenic network with 2.0 times of base flow condition. Units are dyne/cm^2^.**


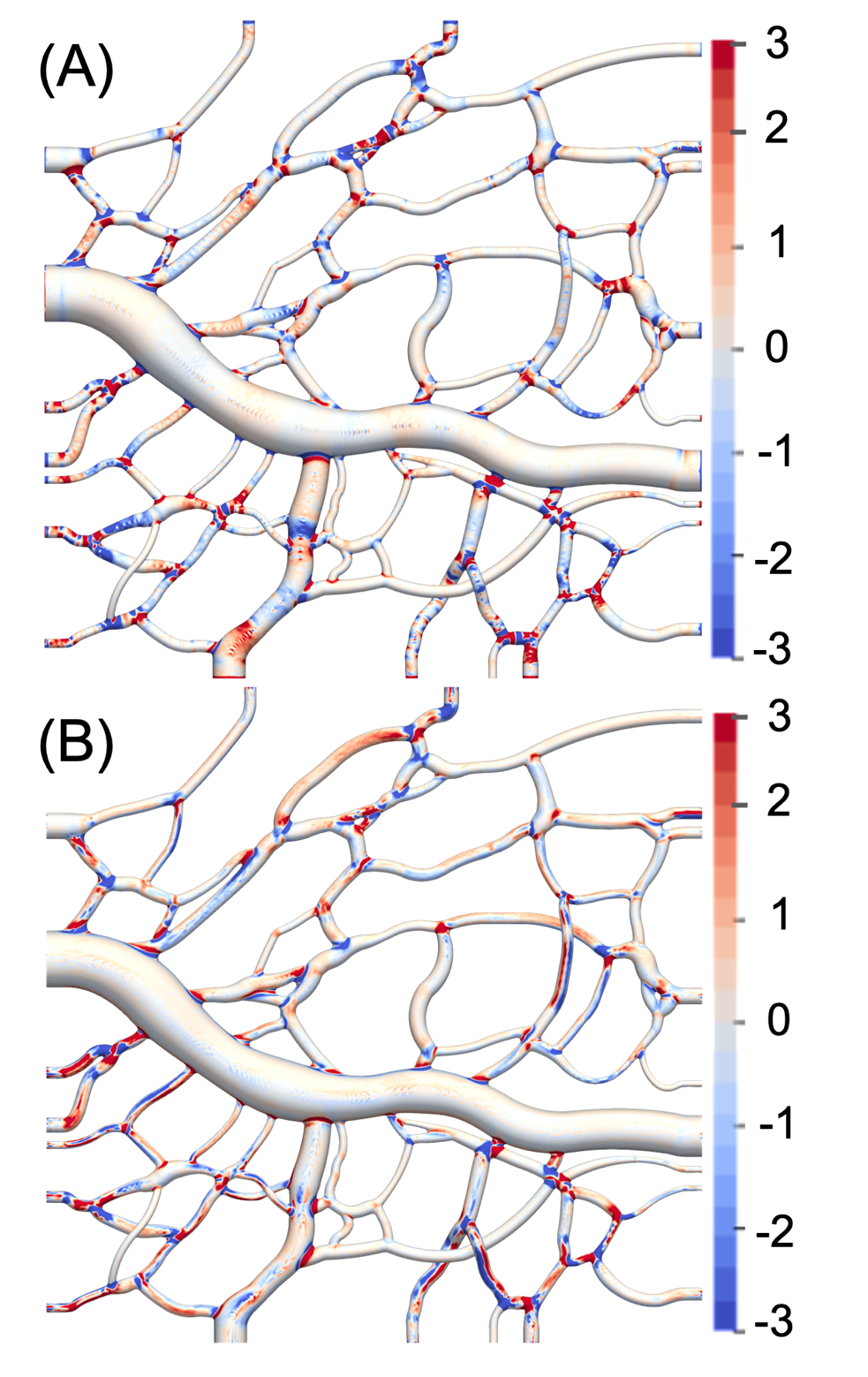


**Figure S13: TAWSSG of the first angiogenic network for base flow condition (A) axial gradient (B) circumferential gradient. Units are dyne/cm^2^/μm.**


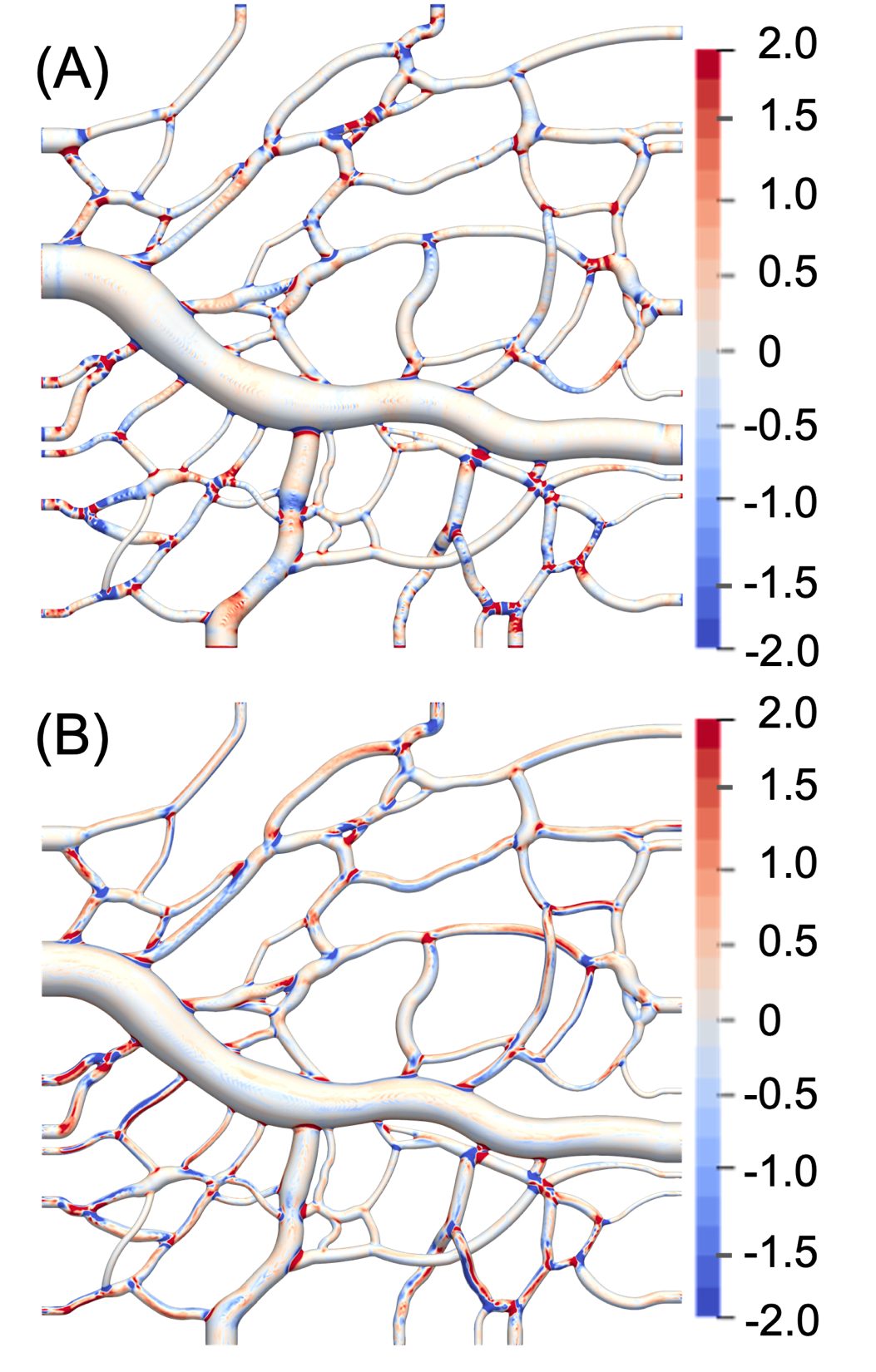


**Figure S14: TAWSSG of the first angiogenic network for 0.5 times of base flow condition (A) axial gradient (B) circumferential gradient. Units are dyne/cm^2^/μm.**


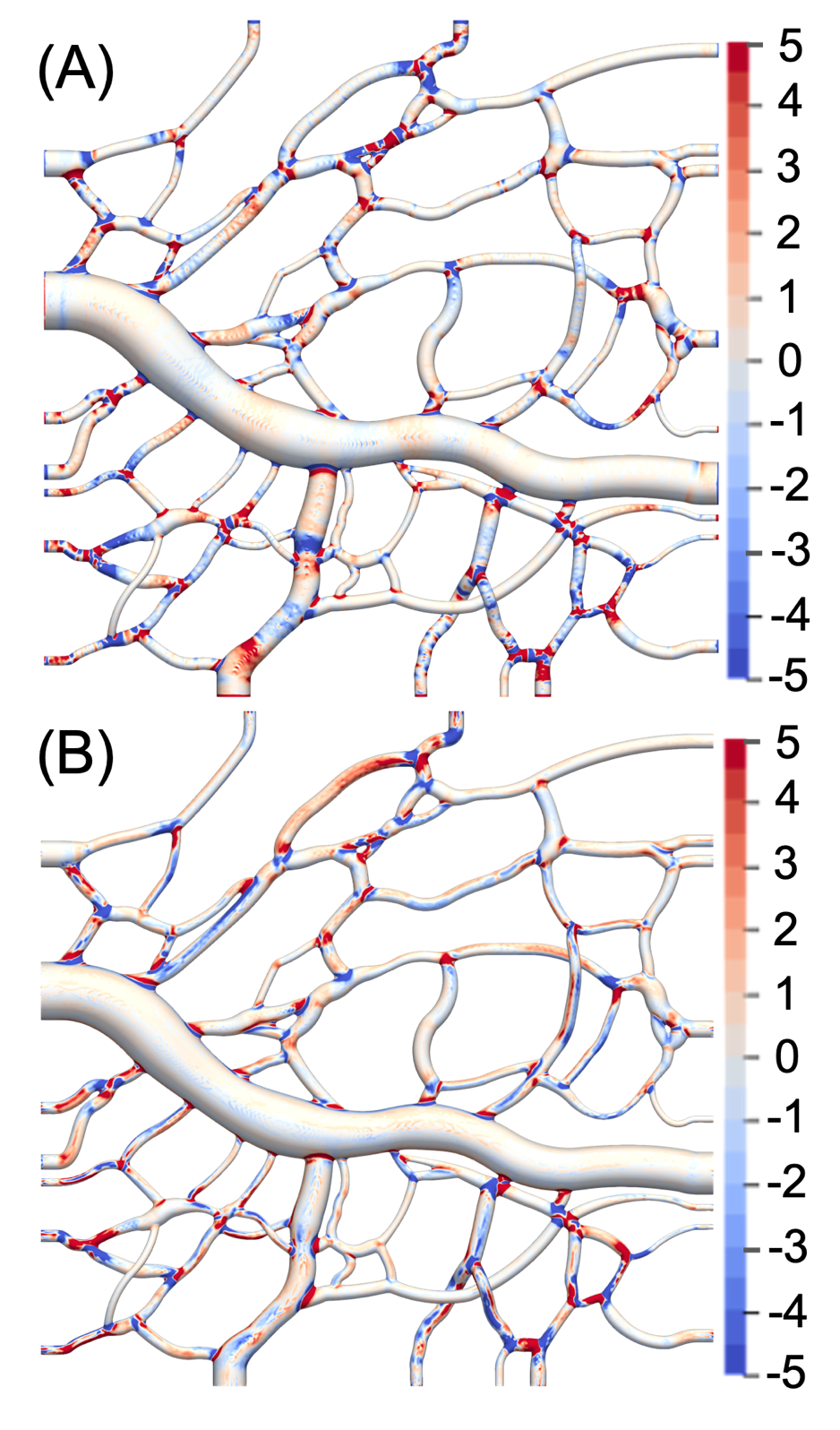


**Figure S15: TAWSSG of the first angiogenic network for 2.0 times of base flow condition (A) axial gradient (B) circumferential gradient. Units are dyne/cm^2^/μm.**


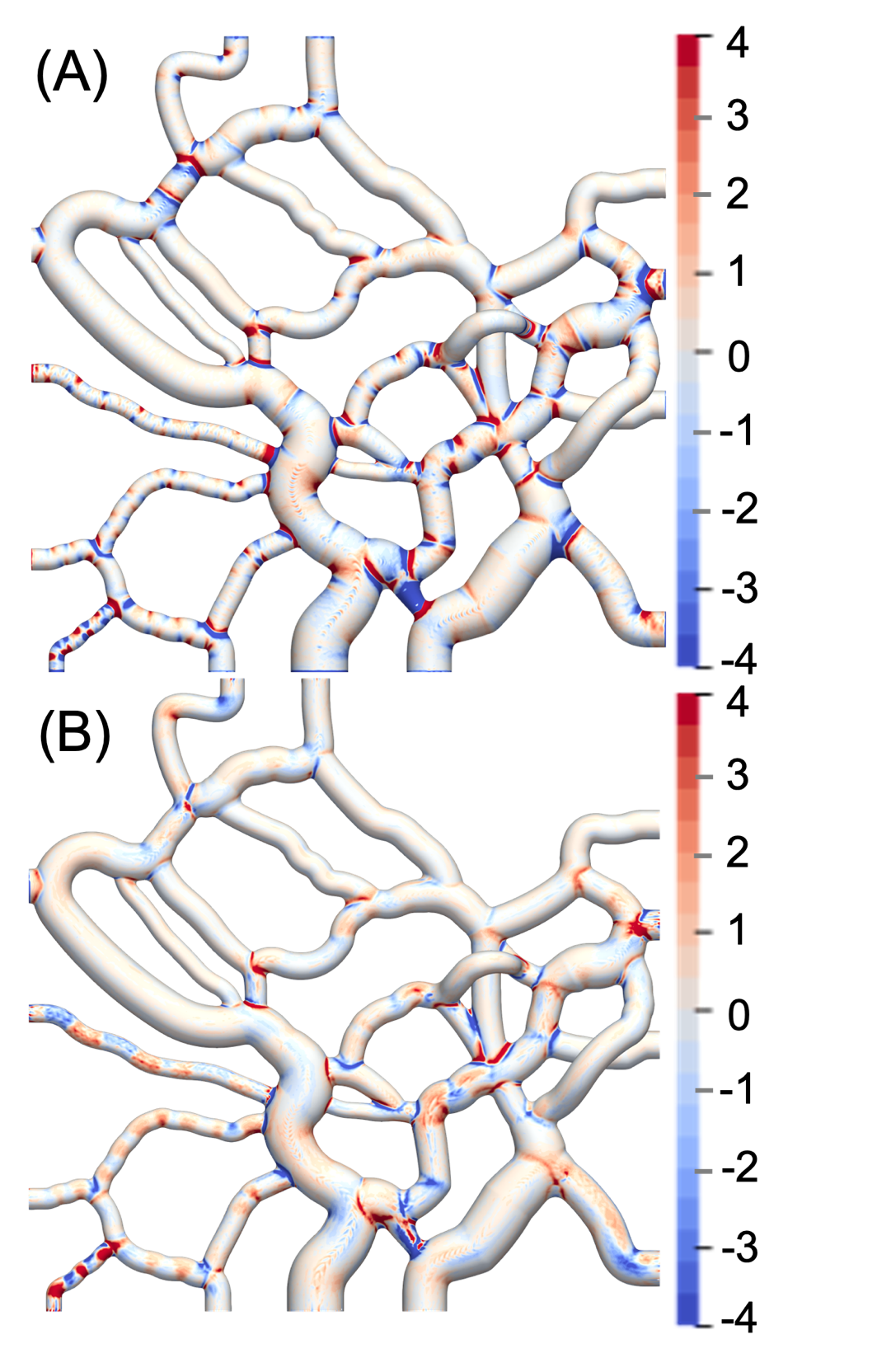


**Figure S16: TAWSSG of the second angiogenic network for base flow condition (A) axial gradient (B) circumferential gradient. Units are dyne/cm^2^/μm.**


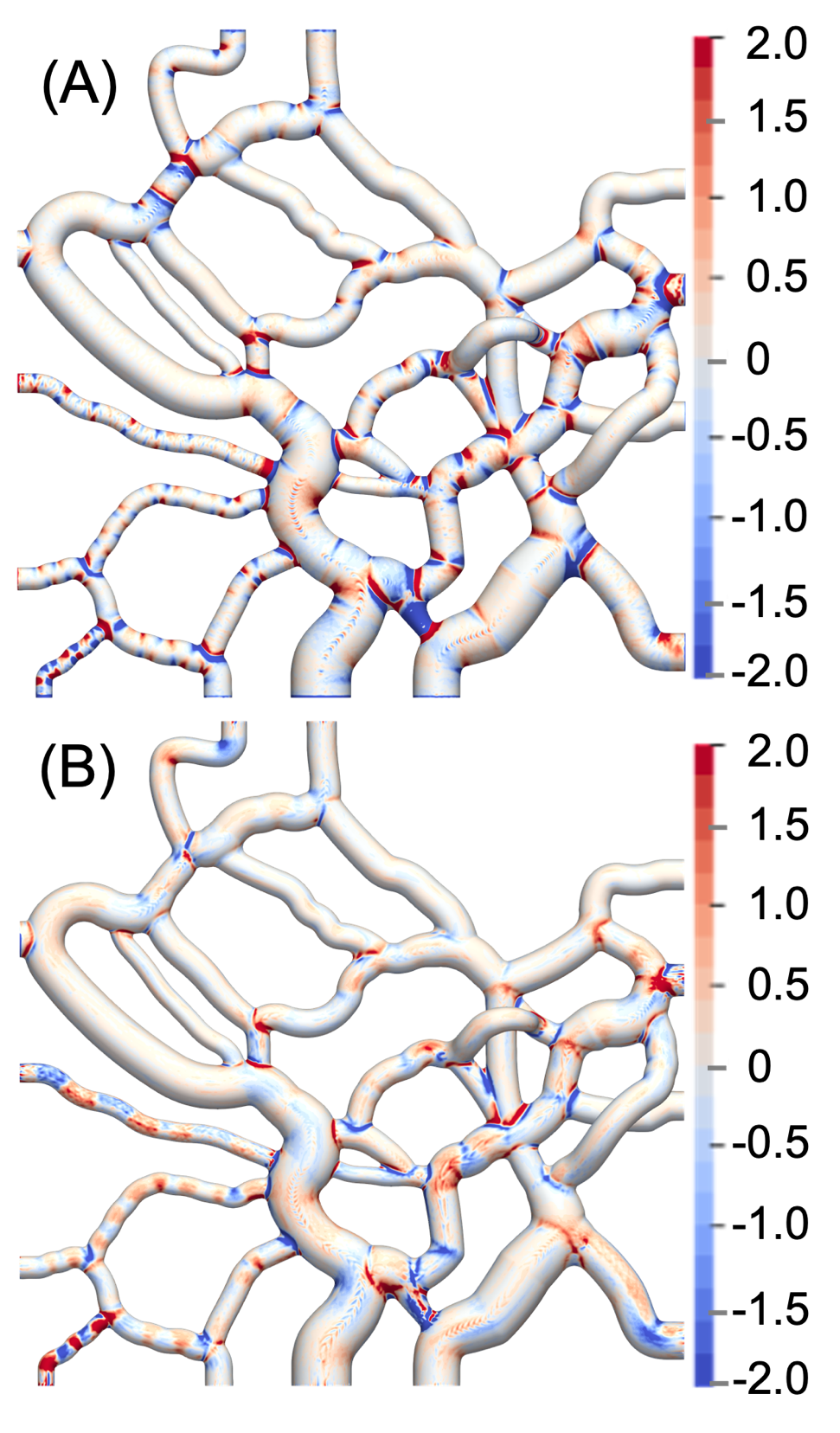


**Figure S17: TAWSSG of the second angiogenic network for 0.5 times of base flow condition (A) axial gradient (B) circumferential gradient. Units are dyne/cm^2^/μm.**


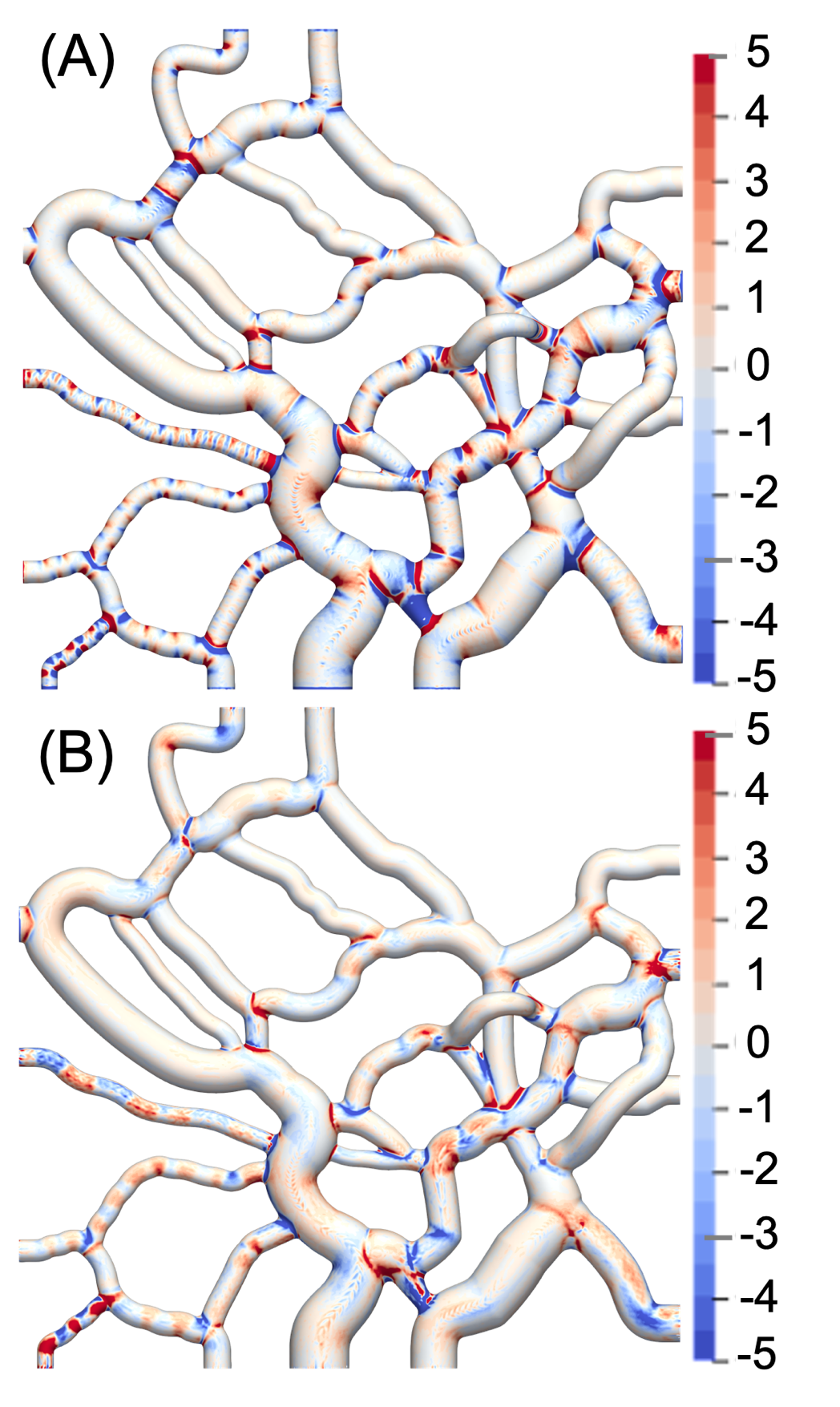


**Figure S18: TAWSSG of the second angiogenic network for 2.0 times of base flow condition (A) axial gradient (B) circumferential gradient. Units are dyne/cm^2^/μm.**

**Flow Rates and Hematocrit at Boundaries, and Hemodynamic Variables Calculated for All Vessels**

The prescribed flow rate boundary conditions at inlet and outlet vessels are based on prescribed shear rates in accordance with literature values from the mesentery, and over a physiological range. Similarly for prescribed hematocrit values maintained in inlet vessels, as described in the main text. We note that in a few vessels, very small flow values were actually prescribed. The reason for this has to do with our method of distributing flow rates among the boundary vessels. The values of flow rates (or, effective shear rates) are initially distributed based on the mentioned range. Then, in order to ensure that the total inflow exactly equals the total outflow, the flows are adjusted from these initial values. We developed an automated routine to do this, which resulted in the small flow rates at the two noted boundaries. In reality, sometimes vessels do have small flow rates for brief periods of time in vivo, and so this does not necessarily represent a non-physiological scenario.

In network 2, the left-most boundary of vessel 57 is modeled as a wall. Originally this was used as an inlet, but while the simulation was running for a brief period it was noticed from the network image that there actually wasn’t an inlet vessel there. It was difficult to discern exactly, but we felt this better represented the network structure. At this point we set the bulk flow at this surface to zero, effectually making it a wall. Given the very small length of this “vessel”, and the minor discontinuity associated with leaving it in, we decided to just continue the simulation rather than starting over. This region is excluded from analysis.

All data predicted by the simulations and used to make the figures in the main text, as well as computer codes can be accessed via the link below. Vessel IDs for each network correspond to those in Figure S19. Executables were compiled on the Lochness HPC linux cluster at NJIT (Intel(R) Xeon(R) Gold 6254 CPU) using the Intel Fortran 2021.4.0 compiler.

**All data generated for this manuscript and computer codes can be accessed** [**here**](https://drive.google.com/drive/folders/1Wb4SpYOM9muX5hjOeOaWaU55lbiPux5C?usp=share_link)**.**


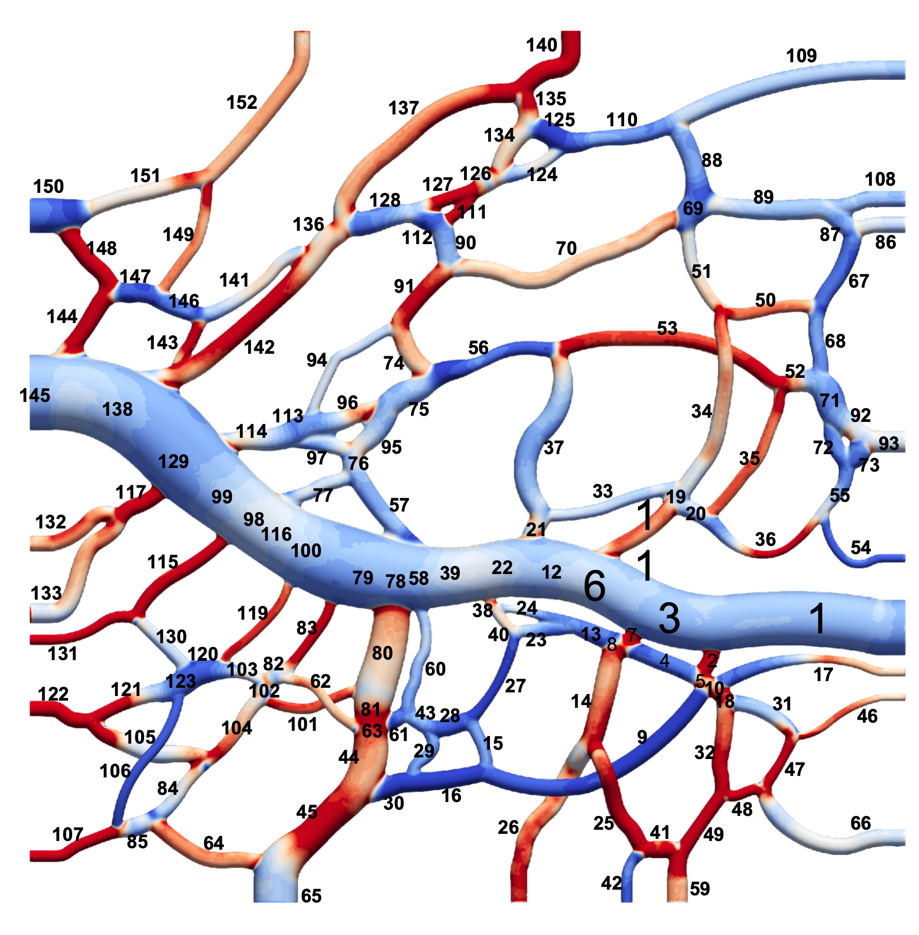


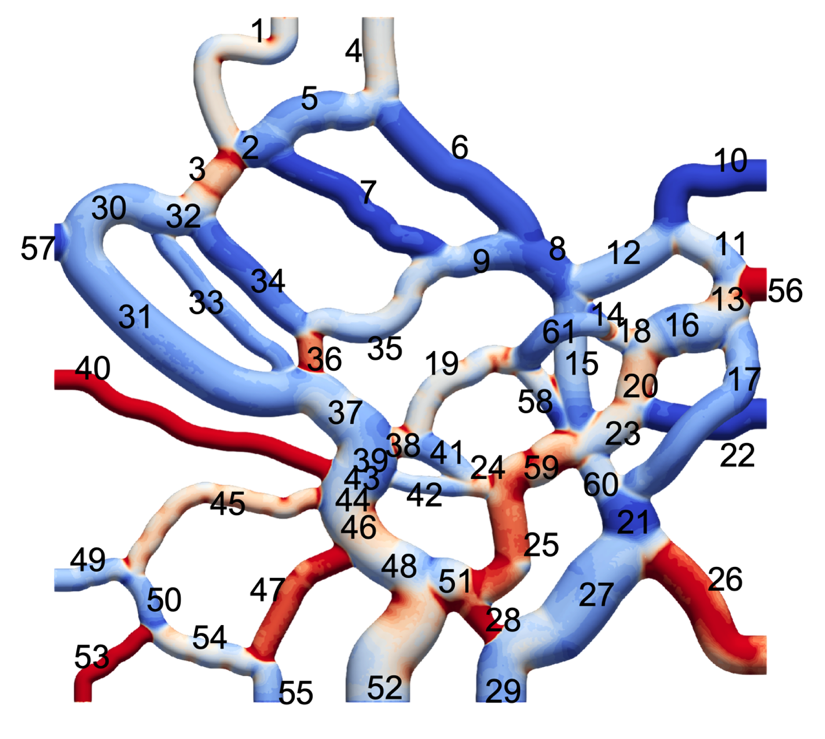


**Figure S19: Vessel IDs for each network**

Oscillation Timescales for WSS metrics based on Fourier Analysis

To quantify the dominant timescale associated with temporal fluctuations in WSS metrics due (i.e. spatial mean, minimum, maximum, and standard deviation), for each vessel an FFT is taken of the time-series data for each vessel. An oscillation timescale is computed for each metric per vessel, by taking the inverse of the dominant frequency. These timescales are plotted in Figure S20 versus vessel diameter, where each data point gives the timescale of the denoted quantity for the vessel.

**Figure S20: Oscillation Timescales for WSS metrics in Each Vessel**

Initial RBC placement, and Injection at Inlets

The initial placement of the RBCs was achieved by packing as many deformed RBCs into each network as was possible. This is done in an automated way, using a comprehensive library of pre-defined deformed RBC shapes, generated from flows in straight tubes. The algorithm essentially goes through each vessel section by section and places as many deformed RBCs as possible without overlaps. We have found this does a good job of achieving initial placements in which deformed RBCs are nicely distributed randomly throughout the networks resembling a realistic physiological scenario.

After RBCs are initialized, simulations begin with the prescribed flow rates at the boundaries. When RBCs cross an outlet boundary, they are removed from the simulation. At inlets, we constantly inject RBCs and attempt to maintain the initial hematocrit level for that region of the vessel. We do this by linking each inlet boundary to a straight tube of equal diameter with deformed RBCs at the desired hematocrit and flow rate. These deformed RBCs are moved into the main simulation, but they cannot be added until there are no overlaps with existing RBCs. Once the local space is clear the RBC is instantiated into the main simulation. We have developed a library of numerous tube diameters and hematocrits to facilitate this linking and injection.

We have found this approach generally works well but is not perfect, as it can be challenging to add RBCs without overlapping with existing RBCs. In some instances this results in a decrease in injected hematocrit, such as the largest inlet vessel on the right side of movie S1. The ability to sufficiently add RBCs just depends on the flow conditions and geometry, and for the network associated with Movie S1, this results in the observed behavior primarily for the main inlet on the right side. We note that while the injected hematocrit reduces, the level is still in a physiological range.

Pressure Contours for All Simulations

Images below provide contours of the time-averaged pressure field for all simulations. While the full pressure field is 3D, data is provided on a slice (plane) through the center of each network to facilitate viewing the field.


**Figure S21: Pressure field on center slice for the first angiogenic network and base flow condition.**

**Figure S22: Pressure field on center slice for the first angiogenic network and 0.5 times base flow condition.**

**Figure S23: Pressure field on center slice for the first angiogenic network and 2.0 times base flow condition.**

**Figure S24: Pressure field on center slice for the second angiogenic network and base flow condition.**

**Figure S25: Pressure field on center slice for the second angiogenic network and 0.5 times base flow condition.**

**Figure S26: Pressure field on center slice for the second angiogenic network and 2.0 times base flow condition.**
